# Phylogeography and genetic effects of habitat fragmentation on endemic *Urophysa* (Ranunculaceae) in Yungui Plateau and adjacent regions

**DOI:** 10.1371/journal.pone.0186378

**Published:** 2017-10-20

**Authors:** Deng-Feng Xie, Min-Jie Li, Jin-Bo Tan, Megan Price, Qun-Ying Xiao, Song-Dong Zhou, Yan Yu, Xing-Jin He

**Affiliations:** 1 Key Laboratory of Bio-Resources and Eco-Environment of Ministry of Education, College of Life Sciences, Sichuan University, Chengdu, China; 2 Sichuan Key Laboratory of Conservation Biology on Endangered Wildlife, College of Life Sciences, Sichuan University, Chengdu, Sichuan, China; National Cheng Kung University, TAIWAN

## Abstract

*Urophysa* is a Chinese endemic genus with only two species (*U*. *rockii* and *U*. *henryi*) distributed in Yungui Plateau (Guizhou Province) and adjacent regions (i.e., Provinces of Hunan, Hubei, Chongqing and Sichuan). The aim of this study was to determine the genetic diversity and population differentiation within *Urophysa* and investigate the effect of the Yungui Plateau uplift and climate oscillations on evolution of *Urophysa*. In this study, micro-morphological characteristics, nine microsatellite loci (SSR), two nuclear loci (ITS and ETS) and two chloroplast fragments (*psb*A-*trn*H and *trn*L-*trn*F) were used to analyze the phylogenetic relationships and assess genetic and phylogeographical structure of *Urophysa*. Isolation by distance (IBD) was performed to research the effects of geographical isolation. We detected high genetic diversity at the species level but low genetic diversity within populations. Striking genetic differentiation (AMOVA) among populations and a significant phylogeographical structure (*N*_ST_ > *G*_ST_, *p* < 0.01) were detected among *U*. *henryi* populations, along with significant effects of isolation by distance (IBD). Molecular clock estimation using calibration strategy and cpDNA substitution rate indicated that the divergence of *U*. *henryi* occurred during late Miocene to early Quaternary, when the orogeny of Yungui Plateau was violent. *U*. *rockii* originated at the early Quaternary and further differentiated at early Pleistocene. Our results suggested that habitat fragmentation played an important role in the genetic diversity and population differentiation of *U*. *rockii* and *U*. *henryi*. Heterogenous geomorphological configuration and complicated environment resulted from rapid uplift of the Yungui Plateau were inferred as important incentives for the modern phylogeograhpical pattern and species divergence of *Urophysa*. The geographical isolation, limited gene flow, specialized morphologies and the Pleistocene climatic oscillation greatly contributed to the allopatric divergence of *U*. *rockii*. Significant genetic drift and inbreeding were detected in these two species, *in situ* measures should be implemented to protect them.

## Introduction

Geological history and climate oscillations are important drivers in the evolution and genetic structure of plant species [1, 2]. Previous researches have indicated that landscape heterogeneity caused by the uplift of the Qinghai-Tibetan Plateau (QTP) greatly contributed to the evolution of many plants (i.e., *Aconitum gymnandrum*, *Taxus wallichiana* and *Rhodiola kirilowii*) in the QTP [3–6]. However, it is uncertain how landscape heterogeneity from uplift in adjacent areas (to the QTP) affected plant evolution [7, 8], such as the Yungui Plateau. The Indian plate has kept moving northward since the Cenozoic, colliding with the Eurasian Plate between 40 and 50 million years ago (Mya). As a result, the orogeny of the QTP is violent, which induced the continuing uplift of the Yungui Plateau, contributing to the formation of its unique geomorphological characteristics and heterogeneous landscape environment [9]. The heterogeneous landscape was typically characterized by low soil water content, periodic water deficiency, and poor nutrient availability, which exert strong selective forces on plant evolution, resulting in remarkably high species richness and endemism in the Yungui plateau [10]. Underlying this species differentiation of the plateau, there was a massive divergence in population genetics and a promotion of ecological diversity. For example, the Yungui Plateau and its adjacent regions have been regarded as an important center of origin for the East Asiatic flora [11]. In addition, the unique characteristics of the plateau have given rise and refuge to a variety of endemic plants [12], and now is a refuge for plants that are threatened elsewhere.

Orogeny always resulted in regions of most plants shifted and divided populations into different spatial-temporal scales, along with breaking up of one patch of habitat into several smaller patches [13, 14]. This phenomenon was called habitat fragmentation, which plays a major role in threatening plant species survival. Habitat fragmentation is often considered to be the main driving force for local species extinction [15, 16] as large populations split into smaller populations with increasing geographic isolation [17]. The spatial isolation of populations may restrict connectivity, resulting in low levels of gene flow between fragments, with subsequently lower genetic diversity and higher genetic differentiation in/among remnant populations [1, 18, 19]. Habitat fragmentation can reduce the fitness of remnant plants by affecting population genetics and dynamics in several ways [19], including genetic erosion [20] population divergence and random genetic drift [1, 21]. Smaller isolated populations often experience greater inbreeding, reduced reproduction rate and offspring survival [22]. Conversely, genetic variation could be maintained or even increased in fragmented populations [23, 24], and habitat fragmentation may play an important role in plant divergence and allopatric speciation [1].

**Fig 1:**
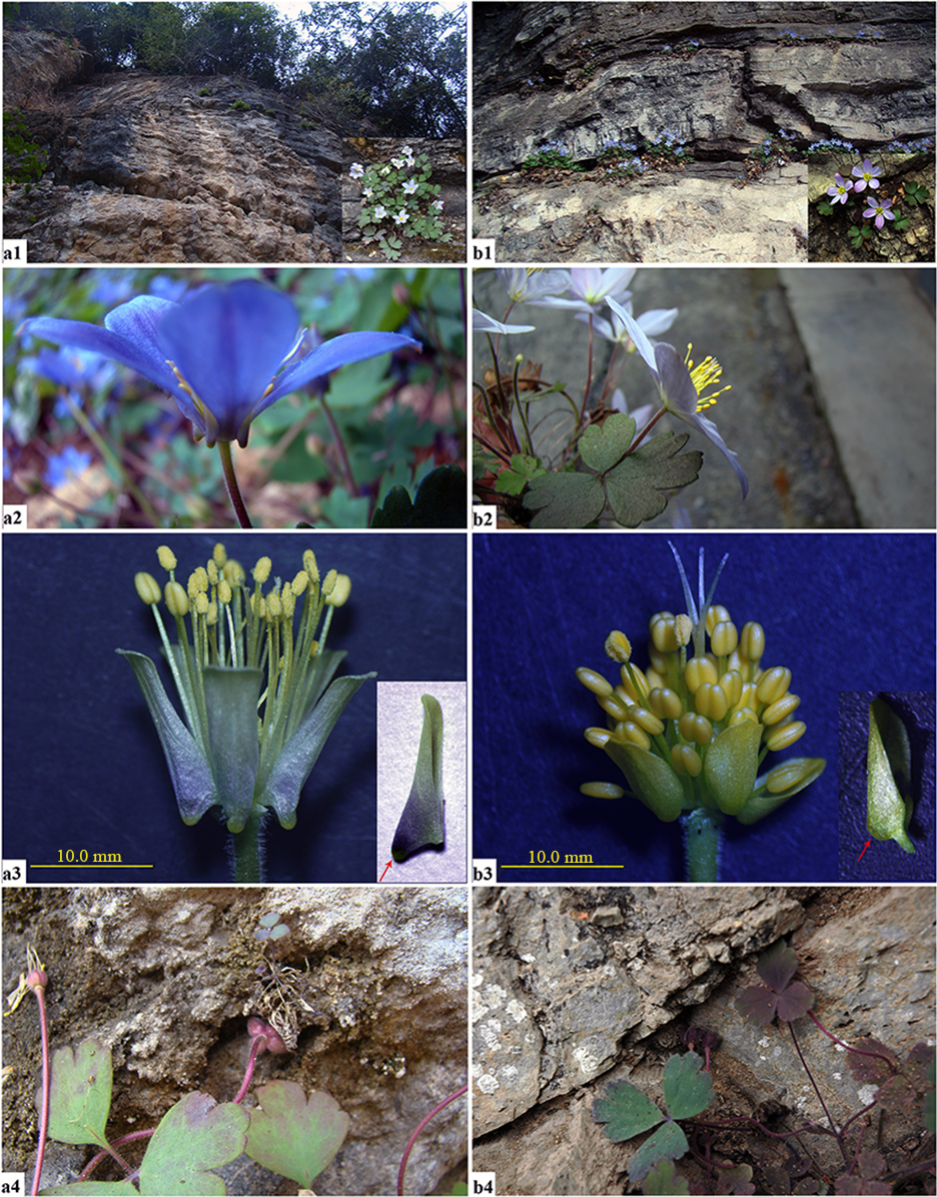
The morphological characters of *Urophysa rockii* (a1–a4) and *Urophysa henryi* (b1–b4). **1**: Habitat feature; **2**: Flowers; **3**: Anatomical characteristics of petals; **4**: Seed dispersal. The red arrows represent spurred petals and saccate petals in **a3** and **b3**, respectively.

*Urophysa* Ulbr. (Ranunculaceae) is an Chinese endemic genus with two species, *U*. *rockii* and *U*. *henryi*. These two species are morphologically distinct: the former has sacs near the base of its petals while the latter has no sac (Fig 1). Additionally, *U*. *rockii* has a more restricted distribution (only located in Jiangyou county, Sichuan Province) than *U*. *henryi* (distributed in Yungui Plateau, south Chongqing, north Hunan and west Hubei). The two species’ natural populations are restricted to small and isolated areas separated by high mountains and deep valleys, and grow in steep and karstic cliffs. The Yungui Plateau is bordered to the east by the Mountains (Mts.) Daba, Wuling and Xuefeng, separating *U*. *henryi* Yungui Plateau populations (populations in Guizhou Province) from neighboring populations (populations in Hunan, Hubei and Chongqing Provinces). Moreover, these two species have strict habitat requirements and are sensitive to environmental change and human activities (scenic spots, power station). By field observations and laboratory experiments, we found that *U*. *rockii* and *U*. *henryi* can not survive once leaving the karst limestone, and populations that located in hydroelectric dams and tourist attractions possess less individuals than that have not been effected by human activities. The plants are collected for Chinese traditional medicine for the treatment of contusions and bruises. However, the population of the two species are in decline [25, 26]. There is ongoing research on the endangered *U*. *rockii*, including its growing environment and conservation strategies [25], biological and ecological characteristics [27, 28] and reproductive biology [29]. However, there have been no studies on the evolutionary history and distribution of these two species. Similarly, species differentiation and the effect of habitat fragmentation in the Yungui Plateau have been little studied. Few studies that have been conducted have found that the occurrence of physical barriers in the Yungui Plateau and its adjacent regions have affect the genetic structure of species such as *Eurycorymbus cavaleriei*, *Saruma henryi* and *Dipentodon* [30–32]. Therefore, we determined the phylogenetic relationship between the two species, their lineage and cause of speciation and why they exhibit such special distribution and distinct morphologies, particularly for the endangered *U*. *rockii*.

Phylogeographical approaches are useful for reconstructing evolutionary histories of species and their close relatives [33], and are reliable for examining interspecific divergence and speciation [1]. Here, we used nine nuclear microsatellites (nSSR), two nuclear gene fragments (ITS and ETS) and two cpDNA regions (*psb*A-*trn*H and *trn*L-*trn*F) to investigate the phylogeography structure of *U*. *rockii* and *U*. *henryi*, and determine their divergence and evolution. It is commonly believed that chloroplast genomes are susceptible to the influence of fragmentation because they are maternally inherited, which can only be dispersed via seeds in sexual reproduction. This is in contrast to nuclear regions, that are diploid and can be dispersed via seeds or pollen. Therefore, we considered that the cpDNA markers were good indicators of population historical dynamics and genetic effects [34]. Our specific aims were to: (1) Evaluate the genetic diversity and population differentiation of *U*. *rockii* and *U*. *henry*. (2) Investigate phylogeographical patterns of *Urophysa* and (3) Investigate the effect of the QTP uplift and climate oscillations on the evolution of *Urophysa*, especially *U*. *rockii*. We believe that this study will be useful for developing conservation strategies and restoration of these two endangered *U*. *rockii* and endemic *U*. *henry*.

## Materials and data

### Plant sampling and morphological observation

A total of 190 individual plants from 14 populations were collected, covering almost the entire geographic range of both species (Table 1), Nine to 16 individual plants were sampled from the 14 populations in the Yungui Plateau (Guizhou Province) and its adjacent regions (i.e., Provinces of Hunan, Hubei, Chongqing and Sichuan Provinces). Healthy leaves were sampled and dried in silica gel until total DNA was extracted. Voucher specimens were deposited in the Herbarium of Sichuan University (SZ). The detailed population information of sampled individuals was measured using a handheld GPS unit. Morphological characteristics were identified using herbarium specimens and fresh materials. The mature pollen grains and leaves dehydrated by graded ethanol were directly mounted on aluminum stubs using conducting carbon adhesive tab, sputter-coated with gold, and then observed using the JSM-7500F scanning electron microscope (SeM, Japan).

**Table 1:** Information for sample collections.

Population code	ID	Sampling locality	latitude (N)	Longitude (E)	Alt. (m)	size
**1**	JY1	^*UR*^Jangyou _(SC)_	31°59′	104°52′	930	11
**2**	JY2	^*UR*^Jangyou _(SC)_	31°59′	104°45′	635	15
**3**	JY3	^*UR*^Jangyou _(SC)_	31°59′	104°51′	943	14
**4**	JY4	^*UR*^Jangyou _(SC)_	31°59′	104°51′	1247	13
**5**	JY5	^*UR*^Jangyou _(SC)_	31°51′	104°35′	1404	12
**6**	SZ1	^*UH*^ Sangzhi_(HN)_	29°40′	110°03′	426	14
**7**	SZ2	^*UH*^Sangzhi _(HN)_	29°39′	109°49′	434	14
**8**	SM	^*UH*^Shimen _(HN)_	29°56′	110°56′	267	15
**9**	YC	^*UH*^Yichang _(HB)_	30°42′	111°17′	245	15
**10**	XY	^*UH*^Xingyi _(GZ)_	25°08′	104°57′	961	12
**11**	ZY	^*UH*^Ziyun _(GZ)_	25°41′	106°05′	1082	16
**12**	AS	^*UH*^Anshun _(GZ)_	26°13′	105°23′	627	14
**13**	SB	^*UH*^Shibing _(GZ)_	27°03′	108°19′	516	16
**14**	CQ	^*UH*^Nanchuan _(CQ)_	30°04′	90°33′	523	9

*UR*:*Urophysa rockii*; *UH*: *Urophysa henryi*; SC = Sichuan province; HN = Hunan province; HB = Hubei province; GZ = Guizhou province; CQ = Chongqing Municipality.

### DNA extraction, sequencing and microsatellite genotyping

Total genomic DNA was isolated from silica-gel dried leaves using plant genomic DNA kit (Tiangen Biotech, Beijing, China). Internal transcribed spacer (ITS) sequences were amplified using the primers ITS4 and ITS5 [35] and ETS fragments were amplified based on the primers ETS9bp and ETS18s [36]. The primers *trn*H and *psb*A [37]were used to amplify the *psb*A-*trn*H sequences, and the *trn*L-*trn*F sequences were amplified by *trn*L and *trn*F [38]. Polymerase chain reaction (PCR) applications were carried out in 30μL reaction volumes. For the ITS and ETS regions, reactions were conducted with the following program: an initial 4-min denaturation at 94°C followed by 30 cycles of 45-sec denaturation at 94°C, 45-sec annealing at 56°C for ITS (53°C for ETS) and 90-sec extension at 72°C with a final 5-min extension at 72°C [35]. For the *psb*A-*trn*H and *trn*L-*trn*F intergenic spacers, the PCR program began with 4-min initial denaturing at 94°C followed by 30 cycles of 1-min denaturation at 94°C, 1-min annealing at 54°C for *psb*A-*trn*H (45 sec at 64°C for *trn*L-*trn*F), and 2-min extension at 72°C. A final extension was run for 7 min at 72°C for *psb*A-*trn*H (5 min at 72°C for *trn*L-*trn*F) [38]. The PCR products were separated in 1.5% (w/v) agarose TAE gel and purified using Wizard PCR preps DNA Purification System (Promega, Madison, WI, USA) following the manufacturer’s instructions. The purified PCR products were sequenced in an ABI Genetic Analyzer (Applied Biosystems Inc., Foster City, CA, USA) in both directions using the PCR primers. The chloroplast DNA sequences and nuclear fragments were edited and assembled using seqMan (DNAstar) [39], then aligned employing CLUSTAL W in MEGA 5 [40] and adjusted manually. All haplotype sequences were deposited in the GenBank database under accession numbers KR820593 to KR820702 (S1 Table).

According to the primers and amplification protocols developed for Li et al. [41], we selected nine pairs of SSR primers for our population genetic data analysis (S2 Table). A pre-experiment was performed using 20 pairs of EST primers, of which, nine pairs of primers that showed significant polymorphism and high homology with relatives *Aquilegia* Li et al. [41] were employed in following analyses (S3 Table). PCR products were separated on 3.5% of agarose gel followed by staining with ethidium bromide. Alleles were sized using PeakScanner v. 1.0 software (Applied Biosystems).

### Data analyses

#### Genetic diversity and divergence

The DnaSP version 5.0 [42] was used to identify different haplotypes and to calculate haplotype diversity (*H*_d_) and nucleotide diversity (*π*). To assess the level of genetic variation and population differentiation, the average within populations gene diversity (*H*_S_), total gene diversity (*H*_T_) and two population differentiation parameters, *G*_ST_ [43] and *N*_ST_ [44] were estimated following the methods described by Pons and Petit [45] using PERMUT [45]. The analysis molecular variance (AMOVA) in Arlequin version 3.5 [46] was used to further quantify genetic differentiation between groups or subgroups, as well as between populations within groups and among individuals within populations. In addition, maximum parsimony median-joining method in NETWORK 4.2.0.1[47] was applied to construct haplotype networks.

Fst is a measure of checking population differentiation and inferring the effects of gene flow and drift [48]. Through constructing the regional scatterplots of the genetic differentiation (quantified as Fst/(1 –Fst)) on geographical distances and calculating the correlation coefficients between them, we can evaluate the relative historical influences of gene flow and genetic drift on regional population structure [49]. Based on this method, the Fst values between pairwise populations were calculated using Arlequin version 3.5 [46]. Scatterplots of the Fst/(1 –Fst) on geographical distances were constructed, and correlation coefficients were calculated along with the significance of correlation in GenAlEx version 6.0 [50], The linearized Fst statistic [Fst/(1 –Fst)] was compared with the matrix of geographical distance by means of a simple Mantel test to detect isolation by distance and to evaluate the relative influences of gene flow and genetic drift on the regional population structure. We used 999 random permutations to test for the Mantel statistical significance.

For SSR analysis, the total number of detected alleles (*N*_A_), allelic richness (*A*_R_) and inbreeding coefficient (*F*_IS_) were calculated using the software fstat 2.9.3.2 [51]. The MICRO-CHECKER v. 2.2.3[52] was used to test for evidence of deviations from the Hardy–Weinberg equilibrium, genotyping errors and null alleles, and compute the expected heterozygosity (*H*_E_) and observed heterozygosity (*H*_O_). A cluster analysis was performed to characterize population structure in STRUCTURE version 2.2 [53], along with 100 000 Markov chain Monte Carlo (MCMC) cycles following 10 000 burn-in cycles, using the admixture model with independent allele frequencies. Eleven replications were performed for each K, in the range K = 2–10, and the optimal K value was determined according to Evanno et al. [54]. In addition, the analysis molecular variance (AMOVA) in Arlequin version 3.5 [46] was used to measure the partitioning of genetic variability within and among populations. In addition, the IBD (isolation by distance) analysis was performed in GenAlEx version 6.0 [50].

#### Phylogenetic analysis

Maximum parsimony (MP) and Bayesian inference (BI) analysis were conducted using the program PAUP* version 4.0 beta 10 [55] and MrBayes v3.1.2 [56], respectively. For parsimony analyses, heuristic searches were carried out with 1000 random sequence replicates, with the tree bisection-reconnection (TBR) branch swapping and the Mul-Trees options selected. All characters were weighted and unordered equally and gaps were treated as missing data. Branch supporting values were estimated with 1000 replicates by bootstrap analysis. For Bayesian inference analyses, the best-fit evolutionary model was selected by the Akaike information criterion (AIC) using MrModeltest 2.2 [57]. The Bayesian Markov chain Monte Carlo (MCMC) searches was performed for 2 × 10^−7^ generations with four chains, sampling trees every 1000 generations and discarding the first 20% of sampled trees as burn-in sample. The remaining trees were used for constructing a 50% majority-rule consensus tree and calculating posterior probability (PP). In addition, maximum likelihood (ML) analyses were performed by RAXML v7.2.8 [58] with 1000 bootstraps under the GTR + G substitution model, Nodes with a bootstrap value of ≥ 95% were considered well-supported in this analysis. Additionally, we made a model test for each locus, and performed the phylogenetic analysis again both in RAxML and MrBayes.

#### Demographic history analysis

Mismatch distribution analysis was undertaken to assess whether genealogy experienced historical population expansions. It is assumed that if populations experienced a sudden demographic expansion should display a unimodal and smooth distribution, and the multimodal distributions are related to demographic equilibrium or decline [59]. Gene flow between populations (*N*_M_) and Neutrality test including the Tajima’s D test [60] and Fu and Li’s D* and F* statistics [61] were calculated by Arlequin version 3.5 [46].

Taking advantage of fossils from *Prototinomiscium vangerowii* (Menispermaceae) [62] and calibration points used in previous studies of *Aquilegia* [63], we estimated the divergence times of *Urophysa*. Bayesian searches for tree topologies and node ages of cpDNA and nrDNA dataset were conducted in BEAST using a GTR + G substitution model selected by JMODELTEST [64] and an uncorrelated lognormal relaxed clock [65]. A Yule process was specified as tree prior and the calibration priors was modelled as normal distributions with a mean time. Unfortunately, no fossils are known for *Urophysa* or any closely related lineages. Therefore, we chose the age of the fossil *Prototinomiscium vangerowii* (Menispermaceae). The Menispermaceae are represented in the Cretaceous of Europe by endocarps assigned to the fossil genus *Prototinomiscium*. Based on the oldest record, from the Turonian of Central Europe, dated to 91.0 (Mya) [62], sets the minimum age of the split between Menispermaceae and Ranunculaceae [66]. In addition, we employed the age interval 51–66 Mya reported by Wikström et al. [67] for the split of *Ranunculus* (subfamily Ranunculoideae) and *Xanthorhiza* (subfamily Coptidoideae), the former genus being in the sister subfamily to Thalictroideae, which includes the genus *Urophysa* [68]. Based on this information, we set priors of 91.0 ± 7.5 and 58.0 ± 2.5 Mya respectively for each calibration point. A Yule speciation model was selected as the tree prior. This is a simple model of speciation that is more appropriate when considering sequences from different species. Considering Bastida *et al*. [63], we dated the origin of Thalictroideae as a mean age of 27.61 Mya with a normal distribution and with a specified 95% confidence interval (CI) of 26.59–28.56 Mya. The stem node of *Aquilegia* was dated to 10.18 Mya with a normal distribution and with the specified 95% confidence interval (CI) of 9.21–11.14 Mya [63]. The program BEAST version 1.8.0 [69] was employed to estimate the divergence of major lineages of *Urophysa*, MCMC runs were performed, each of 2 × 10^7^ generations with sampling every 2000 generations, following a burn-in of the initial 10% cycles. MCMC samples were inspected in Tracer to confirm sampling adequacy and convergence of the chains to a stationary distribution. All of the outgroup sequences used for time calibration were listed in S4 Table.

The substitution rates were also used to estimate time of divergence of *Urophysa*. *Aquilegia incurvata* and *Semiaquilegia adoxoides* were chosen as outgroups [63, 70]. Considering the dominant geographical distribution of cpDNA haplotypes and poorly resolved relationships among nrDNA haplotypes, the cpDNA dataset was used to estimate the divergence time. The best-fit model was GTR + G for cpDNA data inferred from the Akaike information criterion (AIC) with MrModeltest 2.2 [57]. An uncorrelated lognormal model was selected to describe the relaxed clock. The constant cpDNA substitution rates for most angiosperm species have been estimated to be in the range 1–3 × 10^−9^ s/s/y [71]. Given *Urophysa* are perennial plants, the general substitution rates of the plastid sequence (u = 1.52 × 10^−9^ s/s/y) was more suitable. The Markov chain Monte Carlo (MCMC) analyses were run for 2 × 10^7^ generations and trees were sampled every 2000 generations. The first 10% of sampled trees was discarded as the burn-in sample as checked with Tracer 1.5 [69]. The Tree Annotator version1.4.8 [69] was used to summarize the samples in the maximum clade credibility tree with the posterior probability limit set to 0.5. The results were displayed in Figtree version1.3.1 [72].

## Results

### Micro-morphological characteristics

The scanning electron micrographs of leaf epidermis and pollen are shown in S1 Fig. 1) *U*. *henryi* had spindly and numerous epidermal hairs (a1-c1, b2-c2 and a3-c3), while *U*. *rockii* had few hairs that were short and had swollen-bases (d1-d3). 2) Distinctive papillary (a2 and c2) and stelliform (b2) surface ornamentation patterns of leaves were found in *U*. *henryi*, distinctly different to the sinuous surface of *U*. *rockii* (d2). 3) The leaf epidermis in *U*. *henryi* was ornamented by bar-shaped appendages (i.e., b5 and c5 except a5, which was smooth), which was noticeably different from *U*. *rockii* with lineate appendages (d5). 4) The stomatas of *U*. *henryi* can be divided into sunken (a5) and flat types (b5 and c5), but the stomatas of *U*. *rockii* were raised (d5). In the polar view of pollen grains, it is slightly pointed in *U*. *henryi* (e1) but flat in *U*. *rockii* (f1), and the pollen grains pores were sparse in *U*. *henryi* (e2) but numerous in *U*. *rockii* (f2).

### cpDNA sequence analysis

**Fig 2:**
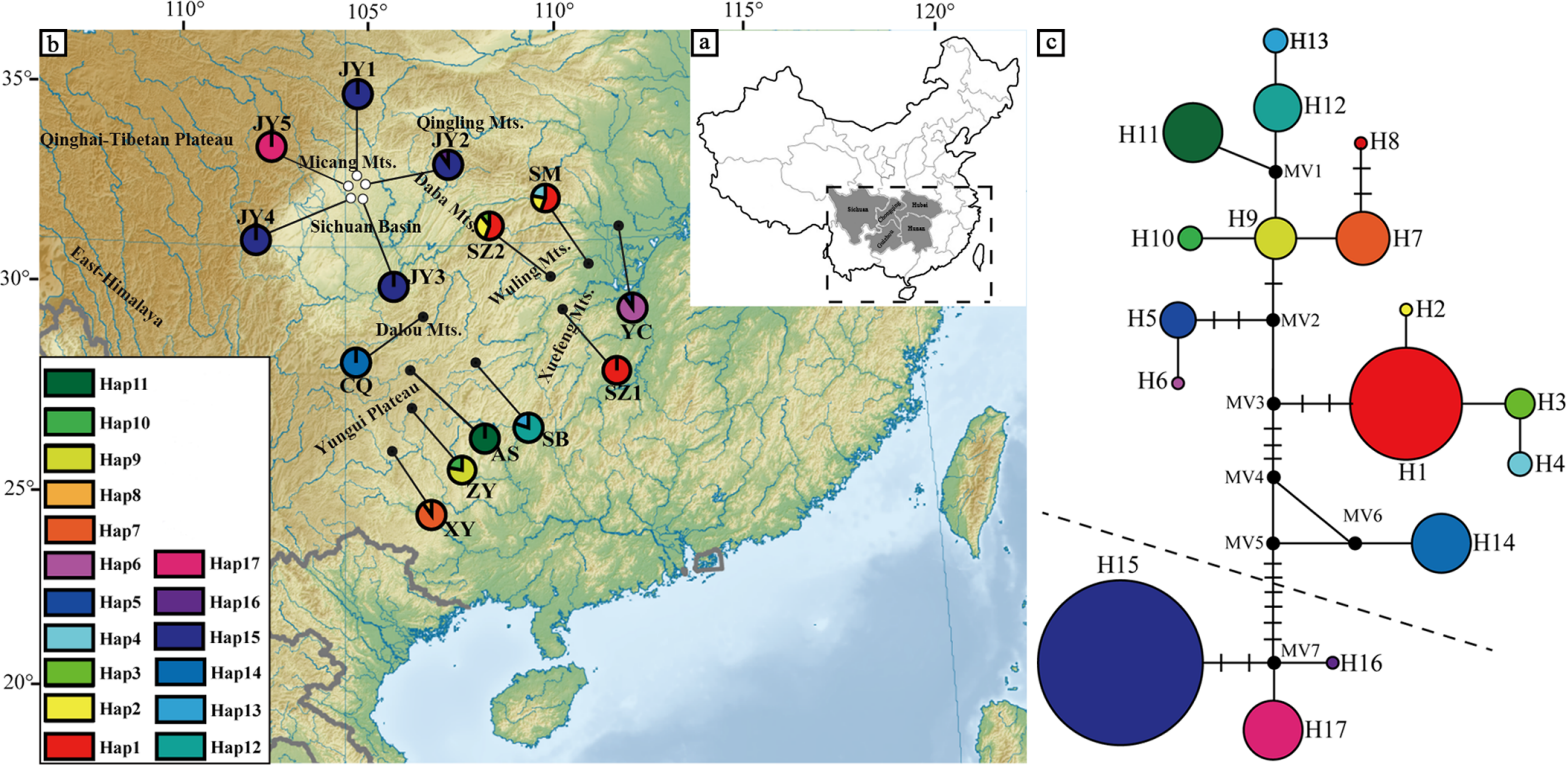
Geographical distribution of cpDNA and NETWORK-derived genealogical relationship. **(a)** Distribution ranges of *U*. *henryi* and *U*. *rockii*. **(b)** The distributions of cpDNA haplotypes (H1–H17) detected in *U*. *rockii* and *U*. *henryi* (population codes see Table 1). **(c)** The statistical parsimony network of cpDNA haplotypes (H1–H17). The topographic map is from the website https://en.wikipedia.org/wiki/File:East_Asia_topographic_map.png.

The two cpDNA regions were aligned along a total length of 1567 bp (*psb*A-*trn*H, 572 bp; *trn*L-*trn*F, 995 bp) and 17 chlorotypes were generated, including 71 polymorphisms (S5 Table). Chlorotypes H1–H14 were found only in *U*. *henryi* and H15–H17 were found in *U*. *rockii* (Fig 2 and Table 2). Only three chlorotypes (H1, H3, H15) were shared by two or more populations and no chlorotype was shared by these two species. In addition, we detected no haplotype located in the center of the chlorotype network, while many private cpDNA haplotypes were detected in each population. At the species level, for *U*. *rockii*, *H*_d_ = 0.742 and *π* = 0.00117, for *U*. *henryi*, *H*_d_ = 0.917 and *π* = 0.01398. Population JY2 of *U*. *rockii* and SM of *U*. *henryi* possessed the highest haplotype diversity and the maximum number of haplotypes. The total genetic diversity (*H*_T_) was higher than the diversity within populations (*H*_S_) for both species, and *H*_T_ and *H*_S_ were higher in *U*. *henryi* than in *U*. *rockii*. Additionally, *N*_ST_ was significantly higher than *G*_ST_ (*N*_ST_ = 0.921, *G*_ST_ = 0.716, *P* < 0.01) but only in *U*. *henryi* (Table 3), indicating a significant phylogeographical structure existing between populations of *U*. *henryi*.

**Table 2:** Haplotypes information of *Urophysa rockii* and *Urophysa henryi*.

Population	cpDNA		nrDNA	
	Haplotype (N)	*H*d (*π*)	Haplotype (N)	*H*d *(π*)
JY1	H15(11)	0.200(0.00056)	H28*(2), H29*(9)	0.200(0.00051)
JY2	H15(12), H16*(3)	0.200(0.00028)	H30*(11), H31*(2), H32*(2)	0.378(0.00034)
JY3	H15(14)	0.000(0.00000)	H33(14)	0.000(0.00000)
JY4	H15(13)	0.000(0.00000)	H33(13)	0.000(0.00000)
JY5	H17*(12)	0.000(0.00000)	H34*(12)	0.000(0.00000)
**Species level**		0.742(0.00117)		0.756(0.00287)
SZ1	H1(14)	0.000(0.00000)	H1*(2), H2*(2), H3*(3), H4*(2), H5*(2), H6*(2), H7*(1)	0.956(0.00765)
SZ2	H1(8), H2*(2), H3(4)	0.639(0.00066)	H8*(8), H9*(6)	0.556(0.00047)
SM	H1(8), H3(4), H4*(3)	0.667(0.00063)	H10*(2), H11*(1), H12*(2), H13*(1), H14*(2), H15*(3), H16*(1), H17*(2), H18*(1)	0.978(0.00659)
YC	H5*(12), H6*(3)	0.200(0.00014)	H19*(15)	0.000(0.00000)
XY	H7*(10), H8*(2)	0.200(0.00042)	H20*(9), H21*(3)	0.356(0.00030)
ZY	H9*(11), H10*(5)	0.389(0.00027)	H22*(3), H23*(13)	0.200(0.00017)
AS	H11*(14)	0.000(0.00000)	H24*(14)	0.000(0.00000)
SB	H12*(13),H13*(3)	0.356(0.00025)	H25*(13), H26*(3)	0.711(0.00076)
CQ	H14*(9)	0.000(0.00000)	H27*(9)	0.000(0.00000)
**Species level**		0.917(0.01398)		0.943(0.00930)

Note: N, individual number

* Exclusive haplotype or sequence types

*H*_d_, Haplotype diversity; *π*, Nucleotide diversity.

**Table 3:** Estimates of gene diversity and interpopulation differentiation.

Species	*H*_S_	*H*_T_	*G*_ST_	*N*_ST_
**cpDNA**				
*U*. *henryi*	0.272 (0.0869)	0.959 (0.0342)	0.716 (0.0996)	0.921(0.0406)
*U*. *rockii*	0.080(0.0490)	0.891(0.1041)	0.910(0.0457)	0.882(0.0289)
**nrDNA**				
*U*. *henryi*	0.397 (0.1288)	0.989 (0.0256)	0.603 (0.1292)	0.832 (0.0990)
*U*. *rockii*	0.116(0.0761)	0.900(0.1170)	0.872(0.0707)	0.951(0.0150)

Average gene diversity within populations (*H*_S_), total gene diversity (*H*_T_), interpopulation differentiation (*G*_ST_), and number of substitution types (*N*_ST_).

AMOVA indicated that 41.32% of the total cpDNA variation was partitioned between species and 57.06% of the variation could be attributed to variation between populations within species (Table 4). For each species, statistically significant variation was detected between populations (88.24% for *U*. *rockii* and 97.30% for *U*. *henryi*). The observed value in the mismatch distribution analysis fitted multimodal curves (S2A–S2E Fig) and the results of Tajima’s D and Fu and Li’s D* and F* statistics were not significantly negative (S6 Table), which indicated that the populations did not undergo expansion. Mantel test results indicated a significant effect of isolation by distance (IBD) at the genus range scale (*r* = 0.322, *p* = 0.001), as well as the species range of *U*. *henryi* (*r* = 0.246, *p* = 0.039), while negative in *U*. *rockii* (*r* = –0.150, *p* = 0.561) (S3A–S3C Fig).

**Table 4:** Analysis of molecular variance (AMOVA) based on the the cpDNA, nrDNA and microsatellite data.

Source of variation	*d*.*f*.	*SS*	*VC*	*PV* (%)	Fixation index
**cpDNA**					
Among species	1	345.133	4.4898	41.32	*F*_SC_ = 0.972^**^
Among populations within species	12	723.856	6.1995	57.06	*F*_ST_ = 0.984^**^
Within populations	177	21.511	0.1763	1.62	*F*_CT_ = 0.413^**^
***U*. *rockii***					
Among populations	4	36.480	0.9	88.24	*F*_ST_ = 0.882^**^
Within populations	61	5.400	0.12	11.76	
***U*. *henryi* populations**					
Among clades	1	275.965	6.055	54.29	*F*_SC_ = 0.943^**^
Among populations Within clade	6	275.574	4.807	43.11	*F*_ST_ = 0.974^**^
Within populations	118	19.711	0.289	2.6	*F*_CT_ = 0.543^**^
***U*. *henryi***					
Among populations	8	715.696	9.339	97.33	*F*_ST_ = 0.973^**^
Within populations	117	19.711	0.256	2.67	
**nrDNA**					
Among species	1	520.627	7.57932	62.14	*F*_SC_ = 0.845^**^
Among populations within species	12	466.071	3.90037	31.98	*F*_ST_ = 0.941^**^
Within populations	177	88.222	0.71725	5.88	*F*_CT_ = 0.621^**^
***U*. *rockii***					
Among populations	4	78.000	1.048	53.11	*F*_ST_ = 0.531^**^
Within populations	61	87.000	0.926	46.89	
***U*. *henryi***					
Among populations	8	388.071	4.91	82.06	*F*_ST_ = 0.821^**^
Within populations	117	83.722	1.073	17.94	
**SSR**					
Among species	1	219.970	1.260	32.0	*F*_SC_ = 0.650^**^
Among populations within species	12	842.490	1.766	44.0	*F*_ST_ = 0.761^**^
Within populations	177	180.500	0.950	24.0	*F*_CT_ = 0.317^**^
***U*. *rockii***					
Among populations	4	78.000	1.048	66.88	*F*_ST_ = 0.669^**^
Within populations	61	87.000	0.926	33.11	
***U*. *henryi***					
Among populations	8	67.995	0.576	66.26	*F*_ST_ = 0.663^**^
Within populations	117	124.243	0.708	33.74	

*F*_CT_ = differentiation among groups; *F*_ST_ = differentiation among populations; *F*_SC_ = differentiation among populations within groups.

** *P* < 0.001, 1000 permutations.

The topological structure of haplotypes derived from Parsimony analyses was similar to that from Bayesian tree and maximum likelihood tree analyses, therefore only the Bayesian tree with maximum parsimony and maximum likelihood bootstrap support values was shown in S4 Fig. In the Bayesian tree, three major clades (Clade I, II and III) corresponded to geographical distributions of the populations. Clade I consisted of haplotypes H7–H13 that were all from the populations of Yungui Plateau, Clade II included the haplotypes H1–H6 mainly located in adjacent regions of Yungui Plateau (Hunan and Hubei Provinces) H14–H17 haplotypes formed the clade III, in which H15–H17 of *U*. *rockii* situated at the edge of Sichuan Basin, were at the crown of phylogenetic tree. The Median-joining network of cpDNA haplotypes was consistent with the strict consensus tree produced by Bayesian analysis (Fig 2C). In addition, after testing the substitution model of each locus and combining to perform phylogenetic analysis, we found that the topology of RAxML and MrBayes trees were similar, and was consistent with topology which was obtained from cpDNA haplotypes (S7 Table and S4 Fig).

**Fig 3:**
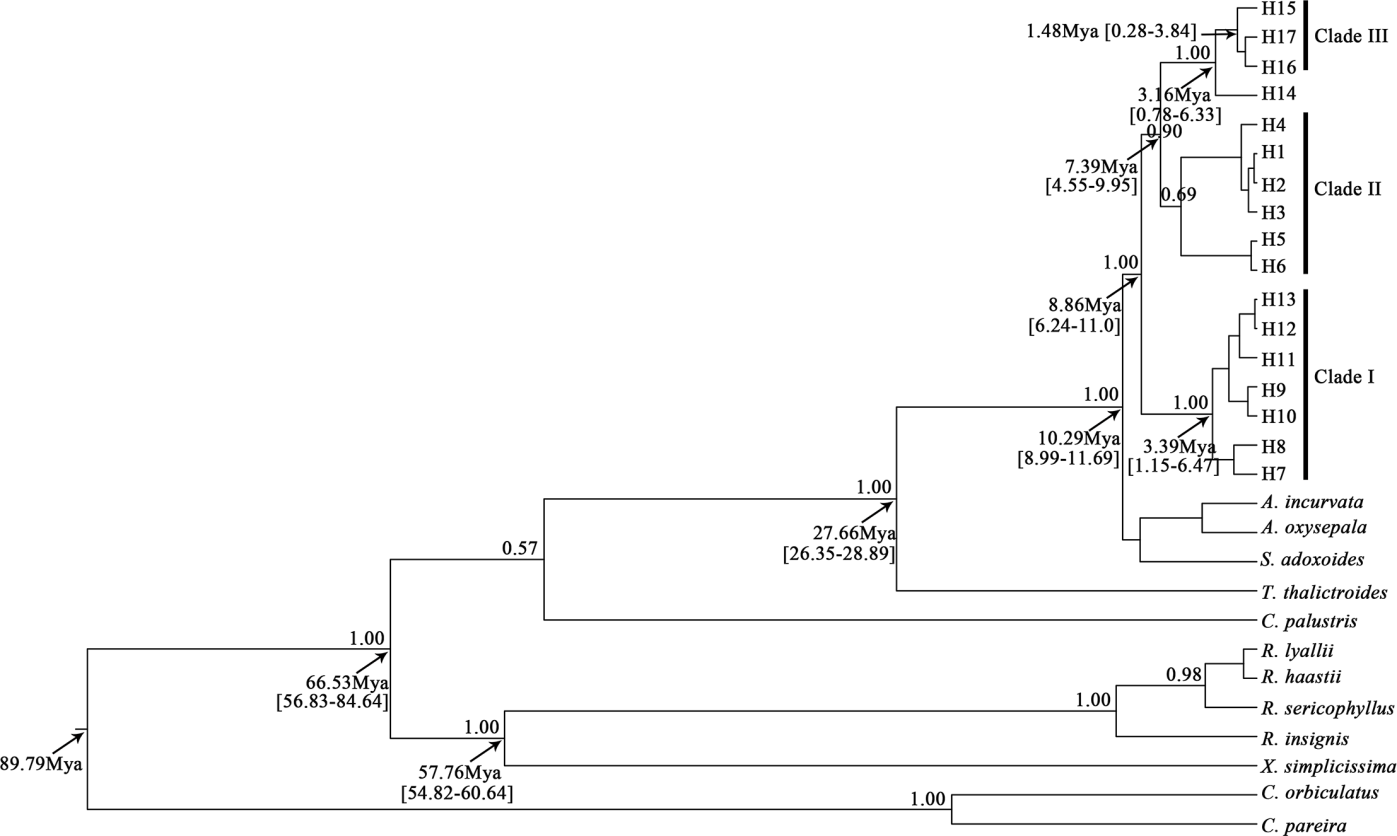
BEAST-derived chronograms for *Urophysa* cpDNA haplotypes. Numbers on the branches indicate the Bayesian posterior probabilities values. Ages of the main clades are shown below the branches. Haplotypes of *U*. *henryi* are H1–H14 and H15–H17 belong to *U*. *rockii*.

From two time-calculated ways (calibration strategy and substitution rate), we estimated the divergence time of *Urophysa*, and found that the calibrated time estimated by cpDNA data is more accurate and reliable compared with time obtained by substitution rate. The origin time of *Urophysa* was dated to approximately 10.29 Mya (95% HPD = 8.99–11.69 Mya) (Fig 3). *U*. *henryi* began to diverge at approximately 8.86 Mya (95% HPD = 6.24–11.00 Mya). However, the origin of *U*. *rockii* did not occur until the early Quaternary, which was dated to approximately 3.16 Mya (95% HPD = 0.78–6.33 Mya), and it diverged at the early Pleistocene, approximately 1.48 Mya (95% HPD = 0.28–3.84 Mya). We obtained a roughly similar time from the substitution rate method, the divergence time of *U*. *henryi* was dated to approximately 7.86 Mya (95% HPD = 4.29–13.07 Mya) and the divergence of *U*. *rockii* was dated to 1.62 Mya (95% HPD = 0.42–3.48 Mya) (S5 Fig).

### nrDNA sequence analysis

**Fig 4:**
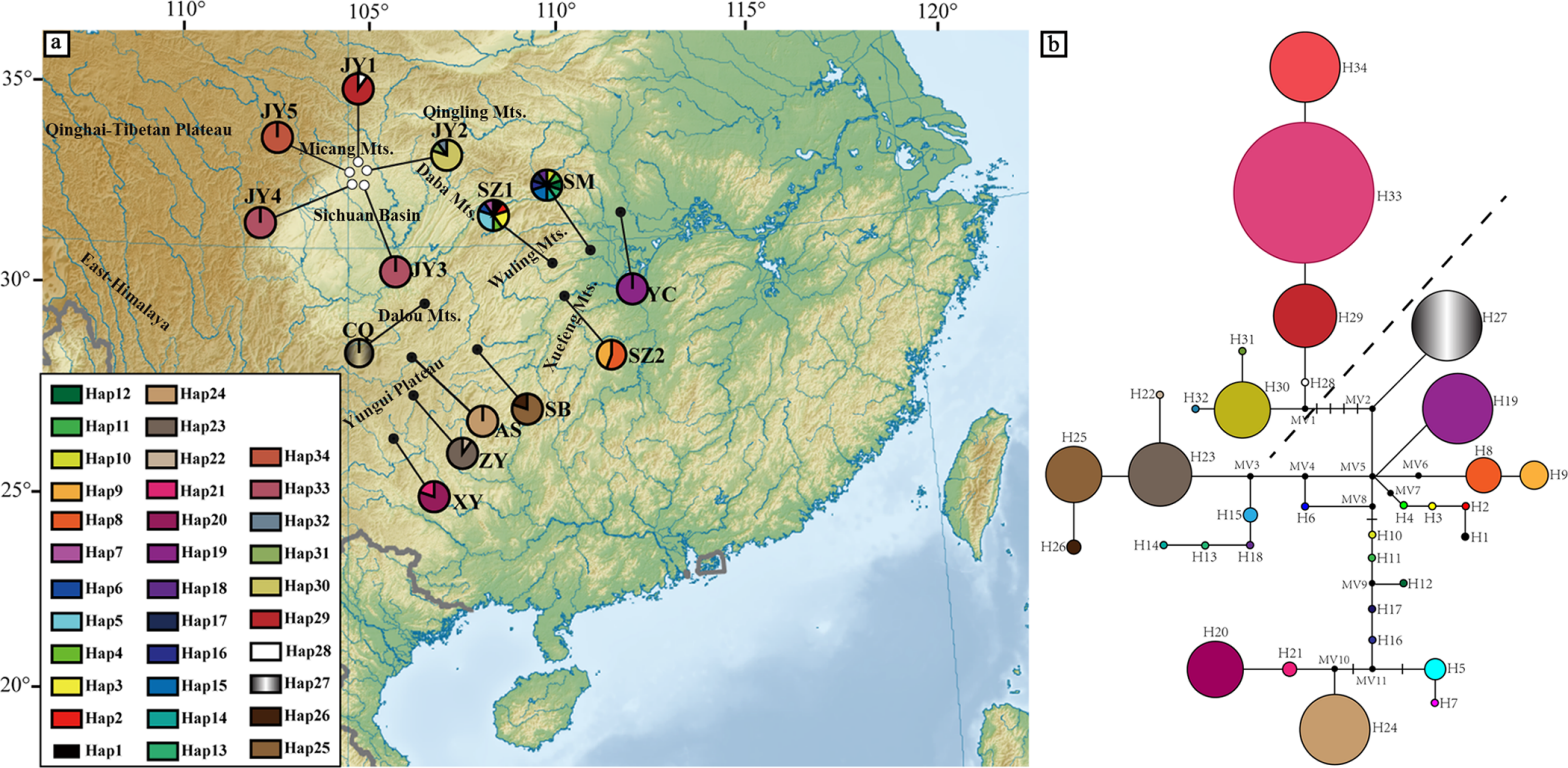
The distributions of nrDNA genotype and Network relationships. **(a)** Geographical distribution of nrDNA haplotypes (H1–H34) detected in *U*. *rockii* and *U*. *henryi* (population codes see Table 1). **(b)** Median-joining network of nrDNA haplotypes (H1–H34). Sizes of the circles in the network are proportional to the observed frequencies of the haplotypes. The small black bars represent mutation steps and the black dots (MV) represent missing haplotypes. The topographic map is from the website https://en.wikipedia.org/wiki/File:East_Asia_topographic_map.png.

The length of aligned sequences was 665 bp for ITS, and 521 bp for ETS. Based on 69 nuclear polymorphic sites combined the two data sets, 34 haplotypes (H1–H34) were detected (Fig 4A and S8 Table). All of the haplotypes were unique to a population except H33 (Table 2), which was shared by population JY3 and JY4. The haplotype diversity (*H*d) ranged from 0 to 0.378 and the nucleotide diversity (*π*) from 0 to 0.00051 within *U*. *rockii*. The haplotype diversity (*H*d) within *U*. *henryi* ranged from 0 to 0.978 and the nucleotide diversity (*π*) from 0 to 0.00765. Diversity at the species level for *U*. *rockii* was *H*_d_ = 0.756 and *π* = 0.00287, and for *U*. *henryi* was *H*_d_ = 0.943 and *π* = 0.00930. The highest haplotype diversity and the maximum number of haplotypes were observed in population SM of *U*. *henryi* and JY2 of *U*. *rockii*.

AMOVA showed that 62.14% of the genetic variation occurred between species and 31.98% of the variation was partitioned among all populations within species. Within both species, variation between populations was significant and accounted for 82.06% and 53.11% of total variation in *U*. *henryi* and *U*. *rockii*, respectively (Table 4). Similar to the findings for cpDNA, mismatch distribution analysis was clearly multimodal (S2F–S2H Fig), as well as a positive and non-significant result of neutrality test (*p* > 0.10) (S6 Table), which indicated the populations did not undergo expansion.

A high total genetic diversity (*H*_T_ = 0.989) and low average within-population genetic diversity (*H*_S_ = 0.397) was detected in *U*. *henryi* and in *U*. *rockii* (*H*_T_ = 0.900, *H*_S_ = 0.116). Similar to cpDNA, the results of nrDNA (*N*_ST_ > *G*_ST_, *P* < 0.01, Table 3) indicated a significant phylogeographical structure existed between populations of *U*. *henryi*. Mantel test results indicated a significant effect of isolation by distance (IBD) at the genus range scale (*r* = 0.301, *p* = 0.004), whereas no significant IBD pattern presented in the two species (S3D–S3F Fig).

Only the Bayesian tree with parsimony bootstrap and maximum likelihood support values was shown in S6 Fig because it has the same topology as the maximum parsimony tree and maximum likelihood tree. *U*. *henryi* and *U*. *rockii* were clustered into a single clade with high bootstrap support and posterior probability values (S6 Fig). The network of nrDNA haplotypes was roughly consistent with the phylogenetic tree, but no ancestral haplotypes were detected (Fig 4B).

Divergence time estimation by nrDNA data (ITS) indicated that *Urophysa* began to diverge at approximately 9.65 Mya (95% HPD = 7.48–11.48 Mya) (S7 Fig), which was slightly older than that by cpDNA data. Estimations of divergence times of species within *Urophysa* are not reliable because of low branches posterior probabilities in the nrDNA tree, and it is better to use the time estimated by cpDNA data.

### Genetic differentiation at the microsatellite loci

A few null alleles were detected by Micro-Checker v.2.2.3 test (Table 5). To test the impact of these null alleles, we removed the microsatellite markers (A41, EST2, EST9) with null alleles in both species and used the remaining six markers to perform the population structure analysis. We found that excluding microsatellite markers with null alleles had little impact on our results (S8 Fig), and *U*. *rockii* and *U*. *henryi* are distinctly separate. Therefore, all microsatellite markers were used for relevant analyses. The results of SSRn indicated low mean allelic richness and gene diversity in *U*. *rockii* (*A*_R_ = 3.386; *H*_O_ = 0.165; *H*_E_ = 0.341) and *U*. *henryi* (*A*_R_ = 5.464; *H*_O_ = 0.249; *H*_E_ = 0.438; see Table 5). All pairwise population genetic differentiations were significant (*p* < 0.001), except between adjacent *U*. *rockii* populations JY3 and JY4 (pairwise F_ST_ = 0.054, *p* > 0.05). Pairwise F_ST_ estimates are listed in S9 Table. *F*_IS_ values ranged from 0.103 to 0.930 across the *U*. *rockii* with an average value of 0.476. *F*_IS_ values ranged from –0.148 to 1.000 for *U*. *henryi*, with an average value of 0.384.

**Table 5:** Characteristics of eight polymorphic microsatellite loci for *U*. *rockii* and *U*. *henryi*.

Species	Locus	*N*_A_	*A*_R_	*H*_O_	*H*_E_	Null alleles present	*F*_IS_	*N*_M_
***U*. *rockii***	A41	6	3.787	0.036	0.407	Y	0.930*	0.763
	B8	2	2.189	0.167	0.251	N	0.234	0.151
	B21	3	2.606	0.091	0.219	Y	0.665*	0.638
	EST1	5	4.110	0.263	0.498	N	0.118*	0.245
	EST2	7	4.505	0.087	0.305	Y	0.720*	0.240
	EST3	4	2.317	0.174	0.232	N	0.220*	0.172
	EST5	5	3.217	0.378	0.474	N	0.103*	0.629
	EST8	6	5.063	0.259	0.481	N	0.461*	0.171
	EST9	4	2.681	0.030	0.198	Y	0.832	0.1766
	**Mean**	4.6	3.386	0.165	0.341		0.476	0.354
***U*. *henryi***	A41	8	5.083	0.116	0.380	Y	1.000*	0.389
	B8	14	7.379	0.173	0.478	N	0.605*	0.310
	B21	6	3.451	0.412	0.474	N	0.104*	0.418
	EST1	8	4.992	0.223	0.404	N	0.204*	0.114
	EST2	10	7.071	0.107	0.448	Y	0.595*	0.302
	EST3	11	3.887	0.342	0.303	N	-0.148	0.424
	EST5	7	4.226	0.096	0.361	N	0.563*	0.108
	EST8	15	8.930	0.297	0.520	Y	0.360*	0.352
	EST9	7	4.16	0.476	0.573	Y	0.169*	0.932
	**Mean**	9.56	5.464	0.249	0.438		0.384	0.372

***N***_**A**_: Observed number of alleles; ***A***_**R**_: Allelic richness; ***H***_**O**_: observed heterozygosity; ***H***_**E**_: expected heterozygosity; **Y/N**: yes/no; ***F***_**IS**_: inbreeding coefficient at the population level

*significant at *P* < 0.05; ***N***_**M**_: Gene flow estimated from *N*_m_ = 0.25*(1-F_ST_)/F_ST_.

**Fig 5:**
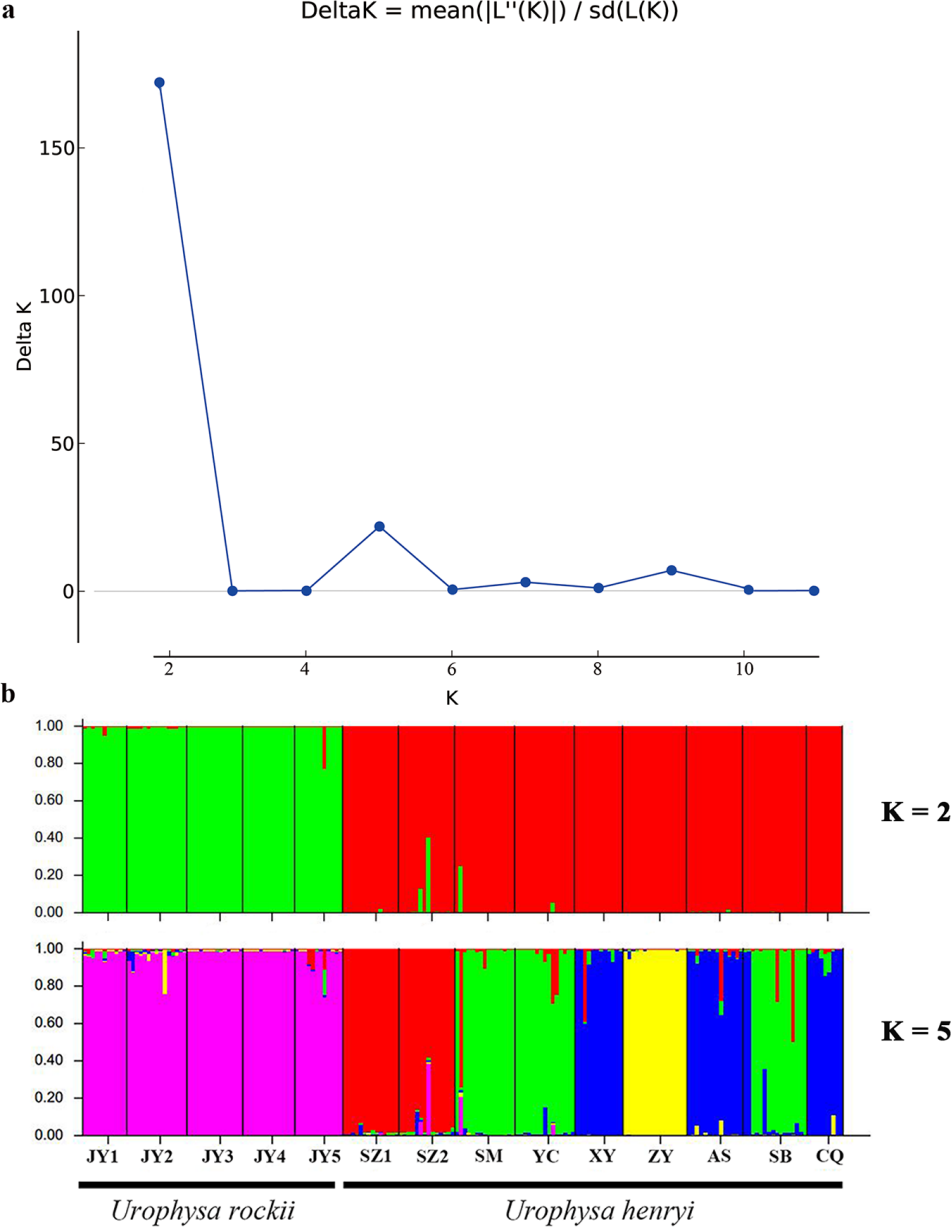
Bayesian clustering analysis for the species of *Urophysa* inferred by STRUCTURE. **(a)** Bayesian inference of the cluster number (K). **(b)** Results for clusters (K = 2 and 5) as detected by STRUCTURE. K was estimated using the distribution of ΔK (second order rate of change of the likelihood distribution). The bars on the figure represent these individuals were sampled from the same species. Bar plots showing Bayesian assignment probabilities. Each vertical bar corresponds to one individual. Populations are separated by black bars and identified at the bottom.

The STUCTURE analysis, using the ΔK method, showed that the optimal K value was K = 2 (Fig 5A), which strongly supported two genetic clusters among our samples and generally corresponded to the two respective species. When K = 5, we detected a small peak, which further divided populations of *U*. *henryi* into four groups (Fig 5). AMOVA indicated that 31.7% of the genetic variation occurred between species (*F*_CT_ = 0.317, *p* < 0.001). Within each species, most of the genetic variation was partitioned among populations (Table 4). A significant effect of isolation by distance (IBD) was detected between the 14 populations of *Urophysa* (*r* = 0.646, *p* = 0.001) and between populations of *U*. *henryi* (*r* = 0.356, *p* = 0.020), while negative in *U*. *rockii* (*r* = –0.175, *p* = 0.484) (S3G–S3I Fig).

## Discussion

### Genetic diversity and significant population differentiation

Based on cpDNA and nrDNA data sets, high haplotype diversity (*H*_d_) and nucleotide diversity (*π*) were observed in *U*. *henryi* and *U*. *rockii* at the species level (Table 2). We detected a high level of total genetic diversity in *U*. *henryi* (*H*_T_ = 0.959 for cpDNA, *H*_T_ = 0.989 for nrDNA) and in *U*. *rockii* (*H*_T_ = 0.891 for cpDNA, *H*_T_ = 0.900 for nrDNA), which was higher than other species of Ranunculaceae, such as *Aconitum gymnandrum* (*H*_T_ = 0.739 for cpDNA) and *Clematis sibirica* (*H*_T_ = 0.496 for nrDNA) [4, 73]. Genetic diversity of species is closely related to environmental factors (e.g., climate, topography) and life history characteristics (i.e., life cycle, breeding system). *U*. *henryi* and *U*. *rockii* possess a relatively long life cycle and sexual reproductive period and high offspring mortality, which may lead to low levels of genetic diversity [74]. It has been found that habitat fragmentation can reduce genetic diversity due to restricted gene flow, genetic erosion and random genetic drift in isolated populations [15, 19, 75, 76]. However, other studies have shown that genetic variation could be maintained or even increased in fragmented populations [23, 24]. Individuals of *U*. *henryi* and *U*. *rockii* are confined to steep and karstic limestones in ravines, which are naturally fragmented. These conditions are found especially in the Yungui Plateau, as high mountains, deep valleys and fast-flowing rivers acting as natural barriers enhancing isolation, drift and mutation [31]. Isolation of *U*. *henryi* and *U*. *rockii* individuals was also supported by the IBD model and Neutrality test (S3 Fig, S6 and S9 Tables), indicating a significant isolation-by-distance pattern between populations of these two species and populations of *U*. *henryi*. Thus, the high genetic diversity observed in these two species may be closely related to their fragmented habitats.

Despite a high level of cpDNA genetic diversity detected at the species level, the average genetic diversity within-population was low (*H*_S_). We found a high coefficient of genetic differentiation (*G*_ST_) in *U*. *henryi* and in *U*. *rockii*, as well as significant phylogeographical structure (*N*_ST_ > *G*_ST_, *P* < 0.01) and variation (*F*_STcpDNA_ = 0.973, *F*_STnrDNA_ = 0.821 for *U*. *henryi*; *F*_STcpDNA_ = 0.882, *F*_STnrDNA_ = 0.531 for *U*. *rockii*) between populations. Additionally, a low level of genetic variation was indicated by microsatellite markers (mean *H*_O_ = 0.249 for *U*. *henryi* and *H*_O_ = 0.165 for *U*. *rockii*) at the population level. While a high degree of genetic differentiation in microsatellites was found in *U*. *henryi* (*F*_ST_ = 0.663) and in *U*. *rockii* (*F*_ST_ = 0.669) (Table 4). These results indicate high genetic differentiation exists between populations. Akin to our study, the combination of low genetic diversity and high genetic differentiation has been reported for other plants in Yungui Plateau and adjacent regions, such as *Cercidiphyllum japonicum*, *Tetrastigma hemsleyanum* and *Cardiocrinum giganteum* [77–79].

Limited gene flow may be a crucial factor resulting in low genetic diversity and high genetic differentiation. It is believed that the breeding system of plant (i.e., flowering and seed proliferation) is an important biological characteristic, which strongly influences the spatial-temporal distribution of genetic variation [80] and affects processes that lead to speciation or extinction [81]. Although the reproduction system and pollination mechanism of *U*. *henryi* has been little studied, previous research of *U*. *rockii* reproduction has indicated the seeds are extremely small and are dispersed into rock gaps due to mechanical strain when the follicle cracks spontaneously after seeds have matured [29]. However, most seeds are washed away by rainwater, and only a few seeds fall to the base of cliff where they may germinate. Petit et al. [82] found that species with seeds dispersed by gravity tended to show higher differentiation between populations than species with wind-dispersed seeds. Interestingly, *U*. *rockii* and *U*. *henryi* are entomophilous plants and bloom in the winter when lower temperatures reduce the visiting frequency of pollinators, which may result in weak pollen flow. The poor gene flow mediated by seeds and pollen was also supported by our results (*N*_M_) (Table 5 and S6 Table). Thus, limited gene flow in such fragmented habitats could lead to significant genetic differentiation between populations [1].

Human activities have significantly influenced the distribution of these two species. Over the past few decades, many hydroelectric dams and tourist attractions have been built in areas where the wild populations of *U*. *henryi* and *U*. *rockii* are located leading to the extensive removal of their habitat. The number of individuals has also sharply declined due to excessive collection for their medicinal value. All of these human activities have reduced the population size, increased fragmentation and isolation, and enhanced population differentiation.

### Demographic history of *U*. *henryi*

The time of origin obtained from the molecular clock estimation is generally congruent with that from points calibration. The molecular clock estimated that the populations of *U*. *henryi* in the Yungui Plateau (Clade I) diverged approximately at 8.86 (6.24–11.0) Mya, which is in accordance with the rapid uplift time of the QTP [83]. It is believed that the QTP reached an elevation similar to the present at about 8.0 Mya, but decreased following extensive faulting. The most recent rapid uplift of the OTP occurred at around 3.6 Mya [84]. Given the geologic close relationship between the Yungui Plateau and the QTP, the orogenic events of the Yungui Plateau were similarly violent during the continuous uplift of the QTP [85]. Therefore, we suggest that rapid QTP uplift and subsequent Yungui Plateau contortions significantly contributed to the differentiation of *U*. *henryi*. Besides, global climate also fluctuated dramatically at that time and the prevailed East Asian monsoon brought plenty of rainfall [86].

In the cpDNA tree, two clades of *U*. *henryi* were revealed (Clade I and clade II) (Fig 3 and S4 Fig), which were consistent with geographical distribution of *U*. *henryi* (Fig 2). This grouping was reflected by a striking differentiation between populations. *U*. *henryi* populations were further divided into four groups when K = 5 in structure analysis, and this roughly corresponded with geographical regions. Mountains such as Wuling, Dalou and Xuefeng. extend from northeast to southwest with an average altitude of 2,000 m [87], and acted as geographical barriers between Yungui Plateau (Clade I) and its adjacent populations (clade II) of *U*. *henryi*. Previous phylogeographical studies have identified that Mt. Wuling and Mt. Xuefeng as the major barriers to gene flow [31, 88]. In addition, the significant increase in geological and ecological variabilities during rapid uplift of the Yungui Plateau has promoted rapid divergence in other small and isolated populations such as *Ligularia*–*Cremanthodium*–*Parasenecio* complex, *Babina pleuraden*, *Dipentodon and Eurycorymbus cavaleriei* [30, 31, 89, 90]. Therefore, the habitat of *U*. *henryi* was fragmented due to geographical barriers and fluctuating climate conditions (i.e., unstable rainfall), and populations of *U*. *henryi* were separated causing high population differentiation.

### The allopatric divergence of *U*. *rockii*

To investigate the phylogenetic relationship between *U*. *rockii* and *U*. *henryi*, we identified distinct morphological traits of petals, leaf epidermis and pollen grains (Fig 1 and S1 Fig). Results of SSRn and nrDNA (Figs 4 and 5, S6 Fig) showed that the *U*. *rockii* and *U*. *henryi* are distinctly separate species and the genus *Urophysa* was a monophyly, which were consistent with previous research [70]. The cpDNA phylogeny demonstrated that *U*. *rockii* was at the end of the cpDNA phylogenetic tree with high bootstrap support values and exhibited significant differentiation with *U*. *henryi*.

Habitat fragmentation is likely to significantly influence the divergence and allopatric speciation of plants [1]. We estimated the origin time of *U*. *rockii* was 3.16 Mya. At that time, orogeny of the Yungui Plateau triggered habitat fragmentation and significant geographic isolation between populations. *U*. *rockii* grows exclusively on cliffs or fissures of rocks in China and only a few populations have been found. Connectivity with *U*. *henryi* populations was hindered by geographic barriers, the Sichuan Basin and Yangzi River, and thus there was no gene flow (Fig 2 and S6 Table). The effect of barriers on gene flow in this region has been documented for *Myotis pilosus* [91] and *Tapiscia sinensis* [92]. The significant isolation caused *U*. *rockii* to undergo allopatric divergence. Additionally, We found considerable genetic differentiation and limited gene flow between *U*. *rockii* and *U*. *henryi* (Table 5 and S6 Table). This high genetic differentiation between populations is likely due to isolated populations with restricted gene flow [93]. This is also supported by the Mantel test between the pairwise F_ST_/(1- F_ST_) and geographic distance (S3 Fig). Moreover, local adaptation may have played an important role in driving allopatric speciation of *U*. *rockii*. As expected, we identified extensive footprints of local adaptation from its specialized morphologies. These specialized morphologies included unusual floral organs such as petaloid sepals that can display various colors in different flower phases, the petals with a nectar spur that could attract more pollinators, and a mass of small seeds (thousand seed weight is 0.6684 ± 0.0038g) [29]. These specialized morphologies likely contributed enhanced reproductive efficiency.

The divergence of the *U*. *rockii* was dated to approximately 1.48 Mya, coinciding with the frequent climatic oscillation during the Pleistocene, which is considered as one of the most important periods for genetic diversification [2]. It is believed that dramatic climate fluctuation during the Pleistocene resulted in massive changes in plant population distributions, with many species shifting to more suitable habitat and other spatial-temporal adjustments [94]. Mismatch analysis indicated that expansion did not occur in populations of *U*. *rockii* (S2E and S2H Fig), and that *U*. *rockii* populations were presumably impacted by the climatic oscillation of the Pleistocene. However, the mountain system located in north of Sichuan Basin was in favor of preserving plant species [95] The mountains of Qinling, Daba and Micang could also have acted as barriers to reduce the effects of the Pleistocene climate [96]. These mountains have also been regarded as a key glacial refuge for other plants including *Pinus massoniana*, *Liriodendron chinense* and *Rhinolophus ferrumequinum* [79, 95, 97]. Overall, we believe that long-term geographical isolation, limited gene flow caused by habitat fragmentation and specialized morphologies probably contributed to the allopatric divergence of *U*. *rockii*. The climatic oscillation of the Pleistocene further promoted population divergence and resulted in the current distribution of *U*. *rockii*.

### Implications for conservation

*U*. *rockii* is an endangered species of China that has a small geographic range, few distinct populations and extremely low germination rate (less than 2%) [29], and each population has a limited number of individuals (all five populations >2,000) [98]. Genetic drift is likely to have occurred, which could have led to the observed genetic diversity decline and increased population differentiation. The correlation estimation of the genetic differentiation [Fst/(1 –Fst)] and geographical distances (isolation by distance) showed that genetic drift was much more influential than gene flow on the distribution of genetic variability. Consequently, populations of *U*. *rockii* are not at equilibrium, which may have resulted from its strict habitat requirement or narrow distribution. A high value of *F*_ST_ indicates high genetic drift load, while a low value of *H*_S_ signifies high inbreeding [22, 99]. In our study, genetic drift and inbreeding were both high (Table 3 and Table 4), which was mirrored by the *F*_IS_ from SSR data (Table 5), indicating a risk of extinction.

Although *U*. *rockii* and *U*. *henryi* demonstrated high genetic diversity at the species level, high genetic drift and inbreeding can be deleterious to species survival. Similarly, species with small population sizes and fragmented distributions are vulnerable to extinction especially with high genetic drift and inbreeding [100]. Protection of *in situ* populations and their habitat, including removing threats (i.e., human disturbance) is essential for *U*. *rockii’s* survival in the wild. *Ex situ* breeding programs can also be implemented using seeds of local provenance to propagate different genotypes to ensure diversity and provide a source of seedling for planting in the wild. Although *U*. *henryi* is not (yet) endangered, frequent human activities have fragmented and degraded its habitat. It is possible that *U*. *henryi* will be threatened in the future due to persistent human disturbance, restricted range and isolated populations. It is necessary to adopt clear and practicable measures to restrict anthropogenic disturbances now, as genetic diversity of wild populations can be maintained and conserved, rather than implement measures after future declines.

## Supporting information

S1 Fig Scanning electron micrographs of leaf epidermis and pollen grains features. Leaf epidermis: *Urophysa henryi* are shwed in **a–c**, *Urophysa rockii* are showed in **d**. In **a–d**, the number after each letter indicate: **1–2**: upper epidermis; **3–5**: lower epidermis. Pollen grains: *U*. *henryi* are showed in **e1–e2**, *U*. *rockii* are showed in **f1–f2**.).
(TIF)

S2 Fig **Mismacth distribution analysis for chloroplast DNA haplotypes (a–e) and nrDNA haplotypes (f–h)**: **(a)** *Urophysa*; **(b)** *U*. *henryi*; **(c)** Clade I; **(d)** Clade II; **(e)** *U*. *rockii* (Clade III); **(f)** *Urophysa*; **(g)** *U*. *henryi*; **(h)** *U*. *rockii*. The solid line represents expected (Exp) values under a sudden population expansion, the dashed line shows observed (Obs) values.
(TIF)

S3 Fig Scatterplots representing relationships between Plots of genetic distance [Fst/(1 –Fst)] and geographic distance (Km). At genus and species levels based on cpDNA **(a**–**c)**, nrDNA **(d**–**f)** and SSR **(g–i)** data. **(a, d, g)** *Urophysa*; **(b, e, h)** *U*. *henryi*; **(d, f, i)** *U*. *rockii*.
(TIF)

S4 Fig Phylogenetic relationships based on the 17 cpDNA haplotypes. Numbers on the branches indicate the maximum Parsimony bootstrap, maximum likelihood support value and Bayesian posterior probabilities, respectively.
(TIF)

S5 Fig BEAST-derived chronograms for *Urophysa* cpDNA haplotype based on constant cpDNA substitution rate (u = 1.52 × 10^−9^ s/s/y). Numbers on the branches indicate the Bayesian posterior probabilities values. Ages of the main clades are shown below the branches. Different colors represent different haplotypes of species: blue, the haplotypes of *U*. *rockii*; red, the haplotypes of *U*. *henryi*.
(TIF)

S6 Fig Phylogenetic relationships of the 34 nrDNA haplotypes. Numbers on the branches indicate the maximum Parsimony bootstrap, maximum likelihood support value and Bayesian posterior probabilities, respectively.
(TIF)

S7 Fig Divergence time calibration for *Urophysa* nrDNA haplotypes. Numbers on the branches indicate the Bayesian posterior probabilities values. Ages of the main clades are shown below the branches. Haplotypes H1–H27 and H28–H34 are possessed by *U*. *henryi* and *U*. *rockii*, respectively.
(TIF)

S8 Fig Histogram of the STRUCTURE analysis for the six microsatellite markers that did not present null alleles. **(a)** Bayesian inference of the cluster number (K). **(b)** Results for clusters (K = 2) as detected by STRUCTURE. The bars on the figure represent these individuals that were retrieved from the same species. Bar plots showing Bayesian assignment probabilities. Each vertical bar corresponds to one individual. Populations are separated by black bars and identified at the bottom.
(TIF)

S1 Table Information of our haplotype sequences deposited in the GenBank. Numbers in the brackets is the sequence number of each haplotype included. *: represent the specific haplotype of each population.
(DOC)

S2 Table Microsatellite markers used in this study. For each primer pair, forward (F) and reverse (R) primer sequence, repeat motif (Repeat), size of cloned allele (bp), optimal PCR annealing temperature (Ta).
(DOC)

S3 Table Homology test of each SSR locus between *Urophysa* and *Aquilegia*. (DOC)

S4 Table psbA-trnH and trnl-trnF sequences of outgroups from Genbank for time calibration. (DOC)

S5 Table Variable sites of the aligned two chloroplast DNA fragments (psbA-trnH and trnL-trnF) among *Urophysa*. (XLS)

S6 Table Parameters of Neutrality test and gene flow among populations. ***N***_**M**_: gene flow.
(DOC)

S7 Table Model test for each of locus and phylogenetic analysis. Numbers on the branches indicate the maximum likelihood support value and Bayesian posterior probabilities, respectively. Different colors represent different populations of species: red, the populations of *U*. *henryi*; blue, the populations of *U*. *rockii*.
(DOC)

S8 Table Variable sites of the aligned ITS and ETS fragments among *Urophysa*. (XLS)

S9 Table Pairwise F_ST_ values among the 14 populations of *U*. *henryi* and *U*. *rockii* based on SSRn data. Note: Values in bold were not significantly different from zero after sequential Bonferroni correction.*: significant at *p* < 0.001.
(DOC)

## References

[1] Young A, Boyle T, Brown T. The population genetic consequences of habitat fragmentation for plants. Trends in Ecology & Evolution. 1996;11(10):413–418. 21237900 10.1016/0169-5347(96)10045-8

[2] Hewitt G. The genetic legacy of the Quaternary ice ages. Nature. 2000;405(6789):907–913. doi: 10.1038/35016000 10879524 10.1038/35016000

[3] Cheng J, Liu XQ, Gao ZJ, Tang DY, Yue JW. Effect of the Tibetan Plateau uplifting on the geological environment of the Yunnan Plateau. Geoscience. 2001;15(3):290–296.

[4] Wang LY, Abbott RJ, Zheng W, Chen P, Wang YJ, Liu JQ. History and evolution of alpine plants endemic to the Qinghai-Tibetan Plateau: *Aconitum gymnandrum* (Ranunculaceae). Molecular Ecology. 2009b;18(4):709–721. 19175501 10.1111/j.1365-294X.2008.04055.x

[5] Liu J, Möller M, Provan J, Gao LM, Poudel RC, Li DZ. Geological and ecological factors drive cryptic speciation of yews in a biodiversity hotspot. New Phytologist. 2013;199(4):1093–1108. doi: 10.1111/nph.12336 23718262 10.1111/nph.12336

[6] Zhang JQ, Meng SY, Rao GY. Phylogeography of *Rhodiola kirilowii* (Crassulaceae): A Story of Miocene Divergence and Quaternary Expansion. PloS One. 2014;9(11):e112923 doi: 10.1371/journal.pone.0112923 25389750 10.1371/journal.pone.0112923 PMC4229298

[7] Meng LH, Yang R, Abbott RJ, Miehe G, Hu TH, Liu JQ. Mitochondrial and chloroplast phylogeography of *Picea crassifolia* (Pinaceae) in the Qinghai-Tibetan Plateau and adjacent highlands. Molecular Ecology. 2007;16(19):4128–4137. doi: 10.1111/j.1365-294X.2007.03459.x 17894761 10.1111/j.1365-294X.2007.03459.x

[8] Li Y, Stocks M, Hemmilä S, Källman T, Zhu H, Zhou Y, et al Demographic histories of four spruce (*Picea*) species of the Qinghai-Tibetan Plateau and neighboring areas inferred from multiple nuclear loci. Molecular Biology and Evolution. 2010;27(5):1001–1014. doi: 10.1093/molbev/msp301 20031927 10.1093/molbev/msp301

[9] Wu FY, Huang BC, Ye K, Fang AM. Collapsed Himalayan-Tibetan orogen and the rising. Tibetan Plateau Acta Petrologica Sinica. 2008;24:1–30.

[10] Kang M, Tao J, Wang J, Ren C, Qi Q, Xiang QY, Huang H. Adaptive and nonadaptive genome size evolution in Karst endemic flora of China. New Phytologist. 2014;202(4):1371–1381. doi: 10.1111/nph.12726 24533910 10.1111/nph.12726

[11] Wang WT. On some distribution patterns and some migration routes found in the eastern Asiatic region. Acta Phytotaxonomica Sinica. 1992;30:1–24.

[12] Li XW, Li J. A preliminary floristic study on the seed plants from the region of Hengduan Mountain. Acta Botanica Yunnanica. 1993;15(3):217–231.

[13] Zhang DF, Fengqu L, Jianmin B. Eco-environmental effects of the Qinghai-Tibet Plateau uplift during the Quaternary in China. Environmental Geology. 2000;39(12):1352–1358.

[14] Sun H, Li ZM. Qinghai-Tibet Plateau uplift and its impact on Tethys flora. Advances In Earth Sciences. 2003;18:852–862.

[15] Aguilar R, Quesada M, Ashworth L, Herrerias-Diego Y, Lobo J. Genetic consequences of habitat fragmentation in plant populations: susceptible signals in plant traits and methodological approaches. Molecular Ecology. 2008;17(24):5177–5188. doi: 10.1111/j.1365-294X.2008.03971.x 19120995 10.1111/j.1365-294X.2008.03971.x

[16] Honnay O, Jacquemyn H. Susceptibility of common and plant species to the genetic consequences of habitat fragmentation. Conservation Biology. 2007;21(3):823–831. doi: 10.1111/j.1523-1739.2006.00646.x 17531059 10.1111/j.1523-1739.2006.00646.x

[17] Lienert J. Habitat fragmentation effects on fitness of plant populations-a review. Journal for Nature Conservation. 2004;12(1):53–72.

[18] Sork VL, Smouse PE. Genetic analysis of landscape connectivity in tree populations. Landscape Ecology. 2006;21(6):821–836.

[19] Heinken T, Weber E. Consequences of habitat fragmentation for plant species: Do we know enough? Perspectives in Plant Ecology, Evolution and Systematics. 2013;15(4):205–216.

[20] Dubreuil M, Riba M, González-Martínez SC, Vendramin GG, Sebastiani F, Mayol M. Genetic effects of chronic habitat fragmentation revisited: strong genetic structure in a temperate tree, *Taxus baccata* (Taxaceae), with great dispersal capability. American Journal of Botany. 2010;97(2):303–310. doi: 10.3732/ajb.0900148 21622391 10.3732/ajb.0900148

[21] Ellstrand NC, Elam DR. Population genetic consequences of small population size: implications for plant conservation. Annual Review of Ecology and Systematics. 1993;24(1):217–242.

[22] Keller LF, Waller DM. Inbreeding effects in wild populations. Trends in Ecology & Evolution. 2002;17(5):230–241.

[23] Carson HL. Increased genetic variance after a population bottleneck. Trends in Ecology & Evolution. 1990;5(7):228–230. 21232361 10.1016/0169-5347(90)90137-3

[24] Xu TT, Abbott RJ, Milne RI, Mao K, Du FK, Wu G, et al Phylogeography and allopatric divergence of cypress species (*Cupressus* L.) in the Qinghai-Tibetan Plateau and adjacent regions. BMC Evolutionary Biology. 2010;10(1):194. 20569425 10.1186/1471-2148-10-194 PMC3020627

[25] Du BG, Zhu DY, Yang YJ, Shen J, Yang FL, Su ZY. Living situation and protection strategies of endangered *Urophysa rockii*. Jiangsu Journal of Agricutural Sciences. 2010;1:324–325.

[26] Xie DF, Zhang L, Hu HY, Guo XL, He XJ. Fragmented habitat drives significant genetic divergence in the Chinese endemic plant, *Urophysa henryi* (Ranuculaceae). Biochemical Systematics and Ecology 2016;69:76–82.

[27] Wang JX, He XJ, Xu W, Meng WK, Su ZY. Preliminary study on *Urophysa rockii*. II. Biological characteristics, ecological characteristics and community analysis. Journal Sichuan Forestry Sciecne Technology. 2011a;32(4):28–39.

[28] Zhang YX, Hu HY, He XJ. Genetic diversity of *Urophysa rockii* Ulbrich, an endangered and rare species, detected by ISSR. Acta Botanica Boreali-Occidentalia Sinica. 2013b;33(6):1098–1105.

[29] Zhang YX, Hu HY, Yang LJ, Wang CB, He XJ. Seed dispersal and germination of an endangered and rare species *Urophysa rockii* (Ranunculaceae). Acta Botanica Boreali-Occidentalia Sinica. 2013c;35(3):303–309.

[30] Yuan QJ, Zhang ZY, Peng H, Ge S. Chloroplast phylogeography of *Dipentodon* (Dipentodontaceae) in southwest China and northern Vietnam. Molecular Ecology. 2008;17(4):1054–1065. doi: 10.1111/j.1365-294X.2007.03628.x 18208489 10.1111/j.1365-294X.2007.03628.x

[31] Wang J, Gao PX, Kang M, Lowe AJ, Huang HW. Refugia within refugia: the case study of a canopy tree (*Eurycorymbus cavaleriei*) in subtropical China. Journal of Biogeography. 2009a;36(11):2156–2164.

[32] Zhou TH, Li S, Qian ZQ, Su HL, Huang ZH, Guo ZG, Zhao GF. Strong phylogeographic pattern of cpDNA variation reveals multiple glacial refugia for *Saruma henryi* Oliv. (Aristolochiaceae), an endangered herb endemic to China. Molecular Phylogenetics and Evolution. 2010;57(1):176–188. doi: 10.1016/j.ympev.2010.07.001 20637294 10.1016/j.ympev.2010.07.001

[33] Hickerson MJ, Carstens BC, Cavender-Bares J, Crandall KA, Graham CH, Johnson JB, et al Phylogeography’s past, present and future: 10 years after Avise. Molecular Phylogenetics and Evolution. 2010;54(1):291–301. doi: 10.1016/j.ympev.2009.09.016 19755165 10.1016/j.ympev.2009.09.016

[34] Petit RJ, Pineau E, Demesure B, Bacilieri R, Ducousso A, Kremer A. Chloroplast DNA footprints of postglacial recolonization by oaks. Proceedings of the National Academy of Sciences USA. 1997;94(18):9996–10001. 10.1073/pnas.94.18.9996 PMC23323 11038572

[35] White TJ, Bruns T, Lee S, Taylor J. Amplification and direct sequencing of fungal ribosomal RNA genes for phylogenetics. PCR protocols: A guide to methods and applications. 1990;18(1):315–322.

[36] Wright SD, Yong CG, Wichman SR, Dawson JW, Gardner RC. Stepping stones to Hawaii: a trans-equatorial dispersal pathway for *Metrosideros* (Myrtaceae) inferred from nrDNA (ITS + ETS). Journal of Biogeography. 2001;28(6):769–774.

[37] Hamilton MB. Four primer pairs for the amplification of chloroplast intergenic regions with intraspecific variation. Molecular Ecology. 1999;8:521–523. 10199016

[38] Taberlet P, Gielly L, Pautou G, Bouvet J. Universal primers for amplification of three non-coding regions of chloroplast DNA. Plant Molecular Biology. 1991;17(5):1105–1109. 1932684 10.1007/BF00037152

[39] Burland TG. DNASTAR’s Lasergene sequence analysis software. Bioinformatics Methods and Protocols. 2000;132:71–91. 10.1385/1-59259-192-2:71 10547832

[40] Tamura K, Peterson D, Peterson N, Stecher G, Nei M, Kumar S. MEGA5: molecular evolutionary genetics analysis using maximum likelihood, evolutionary distance, and maximum parsimony methods. Molecular Biology and Evolution. 2011;28(10):2731–2739. doi: 10.1093/molbev/msr121 21546353 10.1093/molbev/msr121 PMC3203626

[41] Li LF, Ping D, Liao QL, Xiao HX. Genomic and EST microsatellite markers for *Aquilegia flabellata* and cross-amplification in *A*. *oxysepala* (Ranunculaceae). American Journal of Botany. 2011;98(8):213–215. 10.3732/ajb.1100057 21821583

[42] Librado P, Rozas J. DnaSP v5: a software for comprehensive analysis of DNA polymorphism data. Bioinformatics. 2009;25(11):1451–1452. doi: 10.1093/bioinformatics/btp187 19346325 10.1093/bioinformatics/btp187

[43] Nei M. Molecular evolutionary genetics Columbia: Columbia University Press 1987.

[44] Grivet D, Petit RJ. Phylogeography of the common ivy (*Hedera* sp.) in Europe: genetic differentiation through space and time. Molecular Ecology. 2002;11(8):1351–1362. 12144657 10.1046/j.1365-294x.2002.01522.x

[45] Pons O, Petit RJ. Measuring and testing genetic differentiation with ordered versus unordered alleles. Genetics. 1996;144(3):1237–1245. 8913764 10.1093/genetics/144.3.1237 PMC1207615

[46] Excoffier L, Lischer HEL. Arlequin suite ver 3.5: a new series of programs to perform population genetics analyses under Linux and Windows. Molecular Ecology Resources. 2010;10(3):564–567. doi: 10.1111/j.1755-0998.2010.02847.x 21565059 10.1111/j.1755-0998.2010.02847.x

[47] Bandelt HJ, Forster P, Rohl A. Median-joining networks for inferring intraspecific phylogenies. Molecular Biology and Evolution. 1999;16(1):37–48. 10331250 10.1093/oxfordjournals.molbev.a026036

[48] Hutchison DW, Templeton AR. Correlation of pairwise genetic and geographic distance measures: inferring the relative influences of gene flow and drift on the distribution of genetic variability. Evolution. 1999;1898–1914. doi: 10.1111/j.1558-5646.1999.tb04571.x 28565459 10.1111/j.1558-5646.1999.tb04571.x

[49] Rousset F. Genetic differentiation and estimation of gene flow from F-statistics under isolation by distance. Genetics. 1997;145(4):1219–1228. 9093870 10.1093/genetics/145.4.1219 PMC1207888

[50] Peakall R, Smouse PE. GENALEX 6: genetic analysis in Excel. Population genetic software for teaching and research. Molecular Ecology Notes. 2006;6(1):288–295. 10.1093/bioinformatics/bts460 PMC3463245 22820204

[51] Kimura M. A simple method for estimating evolutionary rates of base substitutions through comparative studies of nucleotide sequences. Journal of Molecular Evolution. 1980;16(2):111–120. 7463489 10.1007/BF01731581

[52] Van Oosterhout C, Hutchinson WF, Wills DP, Shipley P. MICRO-CHECKER: software for identifying and correcting genotyping errors in microsatellite data. Molecular Ecology Resources. 2004;4(3):535–538.

[53] Pritchard JK, Stephens M, Donnelly P. Inference of population structure using multilocus genotype data. Genetics. 2000;155(2):945–959. 10835412 10.1093/genetics/155.2.945 PMC1461096

[54] Evanno G, Regnaut S, Goudet J. Detecting the number of clusters of individuals using the software STRUCTURE: a simulation study. Molecular Ecology. 2005;14(8):2611–2620. doi: 10.1111/j.1365-294X.2005.02553.x 15969739 10.1111/j.1365-294X.2005.02553.x

[55] Swofford DL. PAUP*: Phylogenetic analysis using parsimony (*and other methods), Version 4. Sinauer Associates, Sunderland; 2003.

[56] Ronquist F, Huelsenbeck JP. MrBayes 3: Bayesian phylogenetic inference under mixed models. Bioinformatics. 2003;19(12):1572–1574. 12912839 10.1093/bioinformatics/btg180

[57] Nylander JAA. MrModeltest 2.2. Computer program and documentation distributed by the author Evolutionary Biology Centre, Uppsala: Uppsala University; 2004.

[58] Stamatakis A, Hoover P, Rougemont J. A rapid bootstrap algorithm for the RAxML web servers. Systematic Biology. 2008;57(5):758–771. doi: 10.1080/10635150802429642 18853362 10.1080/10635150802429642

[59] Rogers AR, Harpending H. Population growth makes waves in the distribution of pairwise genetic differences. Molecular Biology and Evolution. 1992;9(3):552–569. 1316531 10.1093/oxfordjournals.molbev.a040727

[60] Tajima F. Statistical method for testing the neutral mutation hypothesis by DNA polymorphism. Genetics. 1989;123(3):585–595. 2513255 10.1093/genetics/123.3.585 PMC1203831

[61] Fu YX. Statistical tests of neutrality of mutations against population growth, hitchhiking and background selection. Genetics. 1997;147(2):915–925. 9335623 10.1093/genetics/147.2.915 PMC1208208

[62] Knobloch E, Mai DH. Monographie der Früchte und Samen in der Kreide von Mitteleuropa. Rozpravy ústredního ústavu geologickénho Praha.1986;47:1–219.

[63] Bastida JM, Alcántara JM, Rey PJ, Vargas P, Herrera CM. Extended phylogeny of *Aquilegia*: the biogeographical and ecological patterns of two simultaneous but contrasting radiations. Plant Systematics and Evolution. 2010;284(3–4):171–185.

[64] Posada D. jModelTest: phylogenetic model averaging. Molecular Biology and Evolution. 2008;25(7):1253–1256. doi: 10.1093/molbev/msn083 18397919 10.1093/molbev/msn083

[65] Drummond AJ, Nicholls GK, Rodrigo AG, Solomon W. Estimating mutation parameters, population history and genealogy simultaneously from temporally spaced sequence data. Genetics. 2002;161(3):1307–1320. 12136032 10.1093/genetics/161.3.1307 PMC1462188

[66] Anderson CL, Bremer K, Friis EM. Dating phylogenetically basal eucots using rbcl sequences and multiple fossil reference points. American Journal of Botany. 2005;92:1737–1748. doi: 10.3732/ajb.92.10.1737 21646091 10.3732/ajb.92.10.1737

[67] Wikström N, Savolainen V, Chase M. Evolution of angiosperms: calibrating the family tree. Proceedings of the Royal Society of London B: Biological Sciences. 2001;268(1482):2211–2222. 10.1098/rspb.2001.1782 PMC1088868 11674868

[68] Ro K-E, Keener CS, McPheron BA. Molecular phylogenetic study of the Ranunculaceae: utility of the nuclear 26S ribosomal DNA in inferring intrafamilial relationships. Molecular Phylogenetics and Evolution. 1997;8:117–127. doi: 10.1006/mpev.1997.0413 9299218 10.1006/mpev.1997.0413

[69] Drummond AJ, Rambaut A. BEAST: Bayesian evolutionary analysis by sampling trees. BMC Evolutionary Biology. 2007;7(1):214. 17996036 10.1186/1471-2148-7-214 PMC2247476

[70] Li CY. Classification and Systematics of the Aquilegiinae Tamura. Beijing: The Chinese Academy of Science. 2006;7:3.

[71] Wolfe KH, Li WH, Sharp PM. Rates of nucleotide substitution vary greatly among plant mitochondrial, chloroplast, and nuclear DNAs. Proceedings of the National Academy of Sciences USA. 1987;84(24):9054–9058. 10.1073/pnas.84.24.9054 PMC299690 3480529

[72] Rambaut A. 2009. FIGTREE1.3.1 [online]. Available from http://tree.bio.ed.ac.uk/software/figtree/; 2009.

[73] Zhang HX, Zhang ML, Sanderson SC. Retreating or standing: Responses of forest species and steppe species to climate change in arid Eastern Central Asia. PloS One. 2013a;8(4):e61954. 23596532 10.1371/journal.pone.0061954 PMC3626637

[74] Hamrick JL, Godt MJW. Effects of life history traits on genetic diversity in plant species. Philosophical Transactions Of The Royal Society Of London Series B-Biological Sciences. 1996;351(1345):1291–1298.

[75] Dauber J, Biesmeijer JC, Gabriel D, Kunin WE, Lamborn E, Meyer B, et al Effects of patch size and density on flower visitation and seed set of wild plants: a pan-European approach. Journal of Ecology. 2010;98(1):188–196.

[76] Vranckx GUY, Jacquemyn H, Muys B, Honnay O. Meta-analysis of susceptibility of woody plants to loss of genetic diversity through habitat fragmentation. Conservation Biology. 2012;26(2): 228–237. doi: 10.1111/j.1523-1739.2011.01778.x 22044646 10.1111/j.1523-1739.2011.01778.x

[77] Qi XS, Chen C, Comes HP, Sakaguchi S, Liu YH, Tanaka N, et al Molecular data and ecological niche modelling reveal a highly dynamic evolutionary history of the East Asian Tertiary relict *Cercidiphyllum* (Cercidiphyllaceae). New Phytologist. 2012;196(2):617–630. doi: 10.1111/j.1469-8137.2012.04242.x 22845876 10.1111/j.1469-8137.2012.04242.x

[78] Wang YH, Jiang WM, Comes HP, Hu FS, Qiu YX, Fu CX. Molecular phylogeography and ecological niche modelling of a widespread herbaceous climber, *Tetrastigma hemsleyanum* (Vitaceae): insights into Plio–Pleistocene range dynamics of evergreen forest in subtropical China. New Phytologist. 2015;206(2):852–867. doi: 10.1111/nph.13261 25639152 10.1111/nph.13261

[79] Yang AH, Dick CW, Yao XH, Huang HW. Impacts of biogeographic history and marginal population genetics on species range limits: a case study of *Liriodendron chinense*. Scientific Reports. 2016;6. 10.1038/srep25632 PMC4861920 27162176

[80] Barrett SCH. Mating strategies in flowering plants: the outcrossing-selfing paradigm and beyond. Philosophical Transactions Of The Royal Society Of London Series B-Biological Sciences. 2003;358(1434):991–1004. 10.1098/rstb.2003.1301 PMC1693196 12831464

[81] Holsinger KE. Reproductive systems and evolution in vascular plants. Proceedings of the National Academy of Sciences USA. 2000;97(13):7037–7042. 10.1073/pnas.97.13.7037 PMC34381 10860968

[82] Petit RJ, Aguinagalde I, de Beaulieu JL, Bittkau C, Brewer S, Cheddadi R, et al Glacial refugia: hotspots but not melting pots of genetic diversity. Science. 2003;300(5625):1563–1565. doi: 10.1126/science.1083264 12791991 10.1126/science.1083264

[83] Harrison TM, Copeland P, Kidd WSF, Yin A. Raising Tibet. Science. 1992;255(5052):1663–1670. doi: 10.1126/science.255.5052.1663 17749419 10.1126/science.255.5052.1663

[84] Qiu YX, Li JH, Liu HL, Chen YY, Fu CX. Population structure and genetic diversity of *Dysosma versipellis* (Berberidaceae), a rare endemic from China. Biochemical Systematics and Ecology. 2006;34(10):745–752.

[85] Wang CB, Wang T, Su YJ. Phylogeography of *cephalotaxus oliveri* (cephalotaxaceae) in relation to habitat heterogeneity, physical barriers and the uplift of the yungui plateau. Molecular Phylogenetics and Evolution. 2014;80:205–216. doi: 10.1016/j.ympev.2014.08.015 25160902 10.1016/j.ympev.2014.08.015

[86] An Z, Kutzbach JE, Prell WL, Porter SC. Evolution of Asian monsoons and phased uplift of the Himalaya-Tibetan plateau since Late Miocene times. Nature. 2001;411(6833):62–66. doi: 10.1038/35075035 11333976 10.1038/35075035

[87] Wang ZH, Yang LM, Yang CB. The Study about the Typical Continental Ecosystem of Yunnan-Guizhou Plateau. Beijing: Science Press 2011b.

[88] Dai PF. Phylogeography, phylogeny and genetic diversity of Chimonanthus (Calvcanthaceae). Shanxi, Northwest University (Doctoral dissertation). 2012.

[89] Liu J, Wang Y, Wang A, Hideaki O, Abbott RJ. Radiation and diversification within the *Ligularia*–*Cremanthodium*–*Parasenecio* complex (Asteraceae) triggered by uplift of the Qinghai–Tibetan Plateau. Molecular Phylogenetics and Evolution. 2006;38:31–49. doi: 10.1016/j.ympev.2005.09.010 16290033 10.1016/j.ympev.2005.09.010

[90] Li ZJ, Yu GH, Rao DQ, Yang JX. Phylogeography and demographic history of *Babina pleuraden* (Anura, Ranidae) in southwestern China. PLoS ONE. 2012;7:e34013 doi: 10.1371/journal.pone.0034013 22448286 10.1371/journal.pone.0034013 PMC3309021

[91] Lu GJ, Lin AQ, Luo JH, Blondel DV, Meiklejohn KA, Sun K, et al Phylogeography of the Rickett’s big-footed bat, *Myotis pilosus* (Chiroptera: Vespertilionidae): a novel pattern of genetic structure of bats in China. BMC Evolutionary Biology. 2013;13(1):241. 24188176 10.1186/1471-2148-13-241 PMC4228257

[92] Zhang JJ, Li ZZ, Fritsch PW, Tian H, Yang AH, Yao XH. Phylogeography and genetic structure of a Tertiary relict tree species, *Tapiscia sinensis* (Tapisciaceae): implications for conservation. Annals of Botany. 2015;mcv112. 10.1093/aob/mcv112 PMC4590324 26187222

[93] Shimono Y, Watanabe M, Hirao AS, Wada N, Kudo G. Morphological and genetic variations of *Potentilla matsumurae* (Rosaceae) between fellfield and snowbed populations. American Journal of Botany. 2009;96(4):728–737. doi: 10.3732/ajb.0800242 21628228 10.3732/ajb.0800242

[94] Hewitt GM. Genetic consequences of climatic oscillations in the Quaternary. Philosophical Transactions Of The Royal Society Of London Series B-Biological Sciences. 2004;359(1442):183–195. 10.1098/rstb.2003.1388 PMC1693318 15101575

[95] Qing GF. The study about geographic origin and evolution regularity of *Pinus massonian*a. Journal of Forestry Research. 2002;15:406–412.

[96] Chen DM, Zhang XX, Kang HZ, Sun X, Yin S, Du H, et al Phylogeography of *Quercus variabilis* based on chloroplast DNA sequence in East Asia: multiple glacial refugia and mainland-migrated island populations. PloS One. 2012;7(10):e47268 doi: 10.1371/journal.pone.0047268 23115642 10.1371/journal.pone.0047268 PMC3480369

[97] Flanders J, Wei L, Rossiter SJ, Zhang S. Identifying the effects of the pleistocene on the greater horseshoe bat, *Rhinolophus ferrumequinum*, in east Asia using ecological niche modelling and phylogenetic analyses. Journal of Biogeography. 2011;38(3):439–452.

[98] Wang JX, He XJ. Preliminary study on *Urophysa rockii*. I. Literature research and biological characteristics of *Urophysa rockii*. Journal Sichuan Forestry Sciecne Technology. 2011;32:69–73.

[99] Jaquiéry J, Guillaume F, Perrin N. Predicting the deleterious effects of mutation load in fragmented populations. Conservation Biology. 2009;23(1):207–218. doi: 10.1111/j.1523-1739.2008.01052.x 18847439 10.1111/j.1523-1739.2008.01052.x

[100] Lande R. Anthropogenic, ecological and genetic factors in extinction and conservation. Researches on Population Ecology. 1998;40(3):259–269.

