# Supplementary figures and images for: Phylogeography and genetic effects of habitat fragmentation on endemic Urophysa (Ranunculaceae) in Yungui Plateau and adjacent regions
Source: PLoS One. 2017 Oct 20;12(10):e0186378. doi: 10.1371/journal.pone.0186378 (PMC5650156; doi:10.1371/journal.pone.0186378)

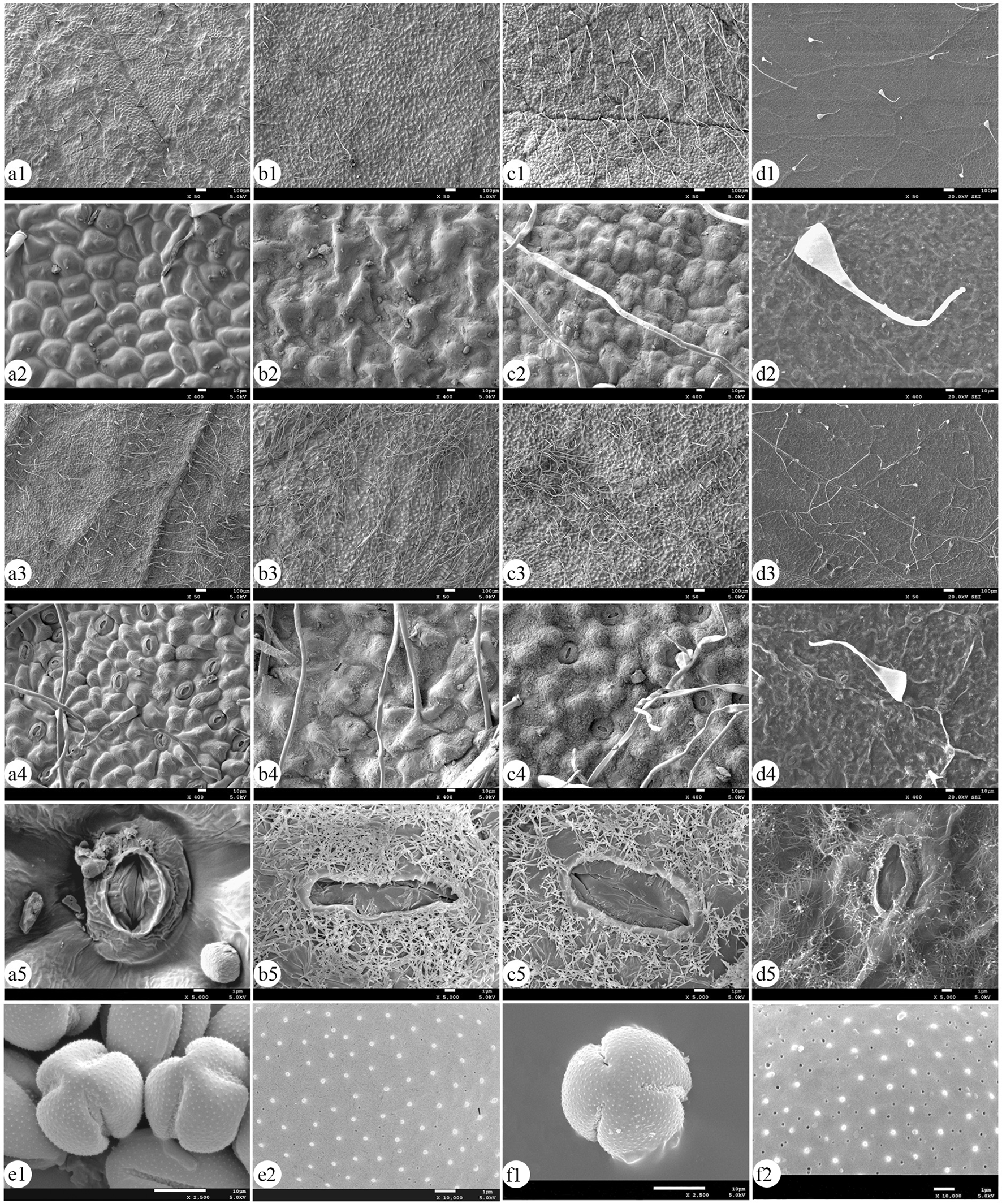

Supplement: S1 Fig — Leaf epidermis: Urophysa henryi are shwed in a–c, Urophysa rockii are showed in d. In a–d, the number after each letter indicate: 1–2: upper epidermis; 3–5: lower epidermis. Pollen grains: U. henryi are showed in e1–e2, U. rockii are showed in f1–f2.). (TIF) [file pone.0186378.s001.tif]

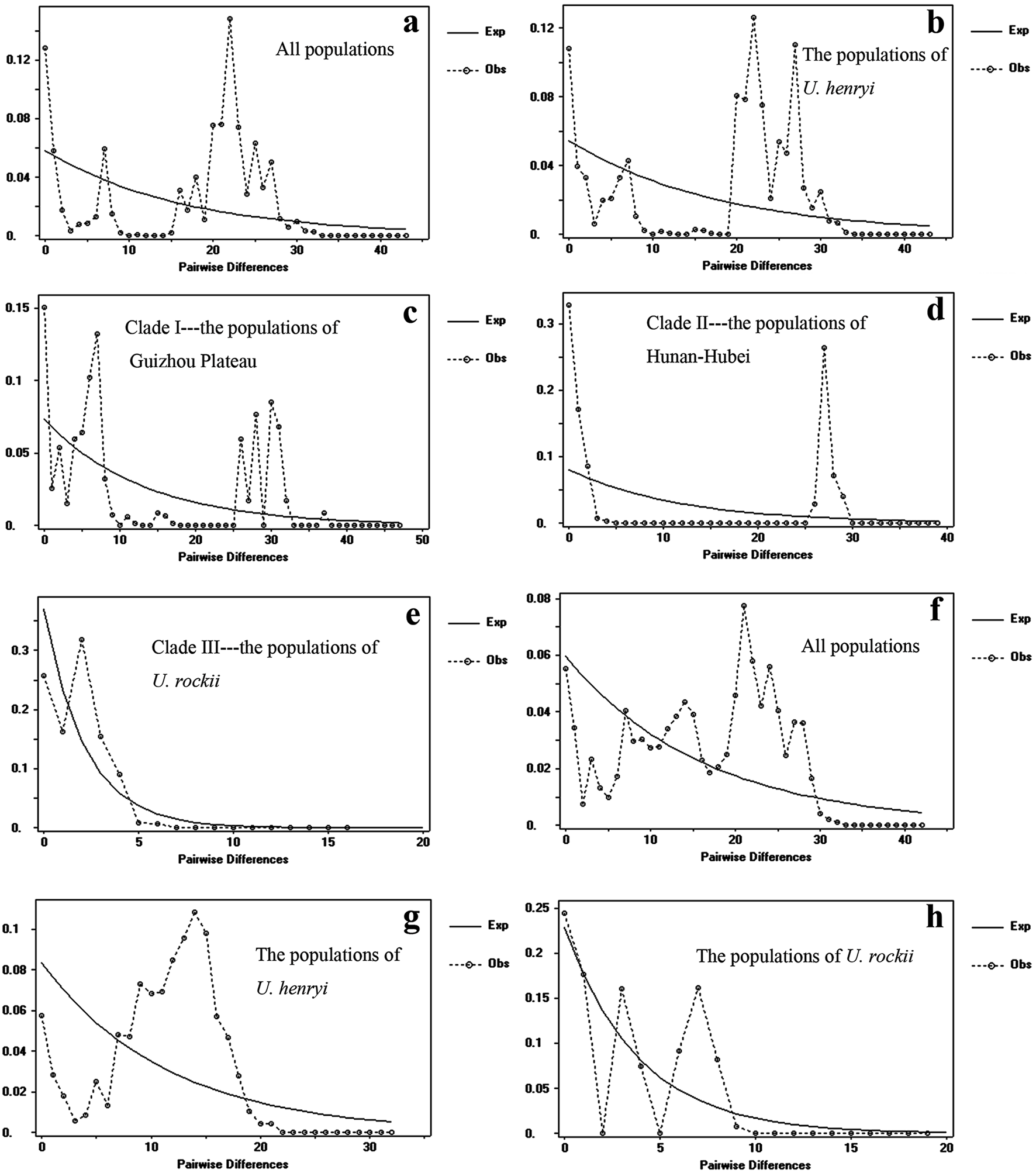

Supplement: S2 Fig — Mismacth distribution analysis for chloroplast DNA haplotypes (a–e) and nrDNA haplotypes (f–h): (a) Urophysa; (b) U. henryi; (c) Clade I; (d) Clade II; (e) U. rockii (Clade III); (f) Urophysa; (g) U. henryi; (h) U. rockii. The solid line represents expected (Exp) values under a sudden population expansion, the dashed line shows observed (Obs) values. (TIF) [file pone.0186378.s002.tif]

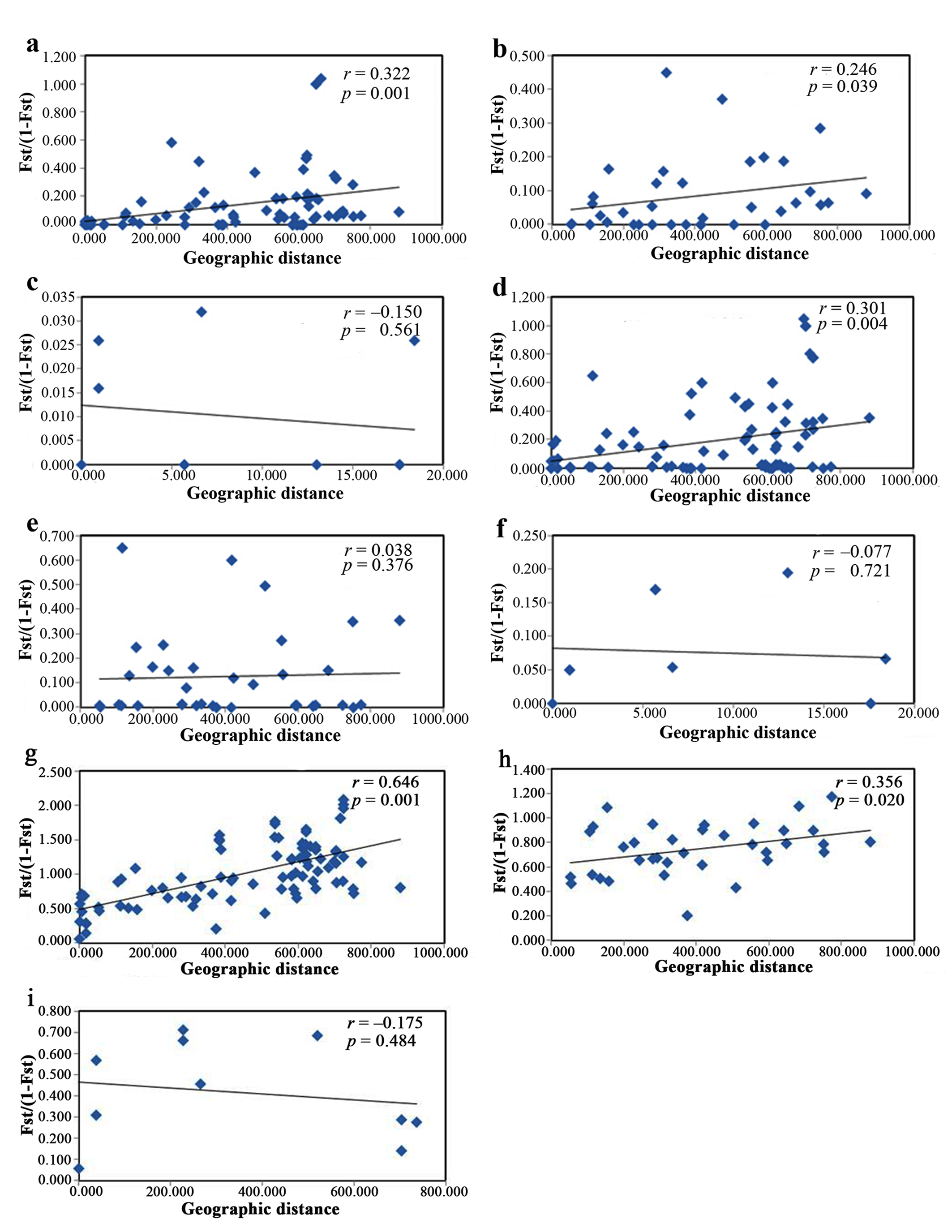

Supplement: S3 Fig — At genus and species levels based on cpDNA (a–c), nrDNA (d–f) and SSR (g–i) data. (a, d, g) Urophysa; (b, e, h) U. henryi; (d, f, i) U. rockii. (TIF) [file pone.0186378.s003.tif]

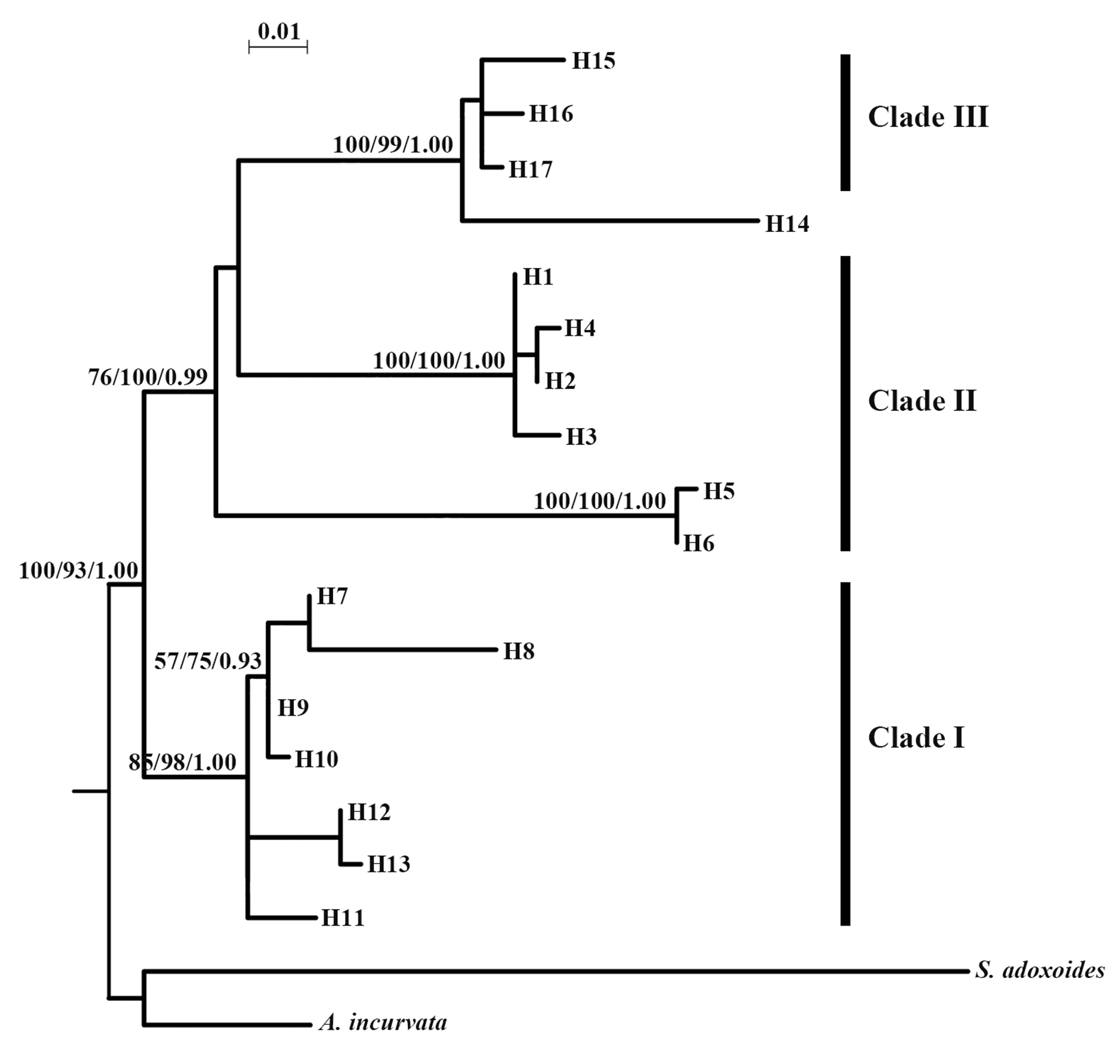

Supplement: S4 Fig — Numbers on the branches indicate the maximum Parsimony bootstrap, maximum likelihood support value and Bayesian posterior probabilities, respectively. (TIF) [file pone.0186378.s004.tif]

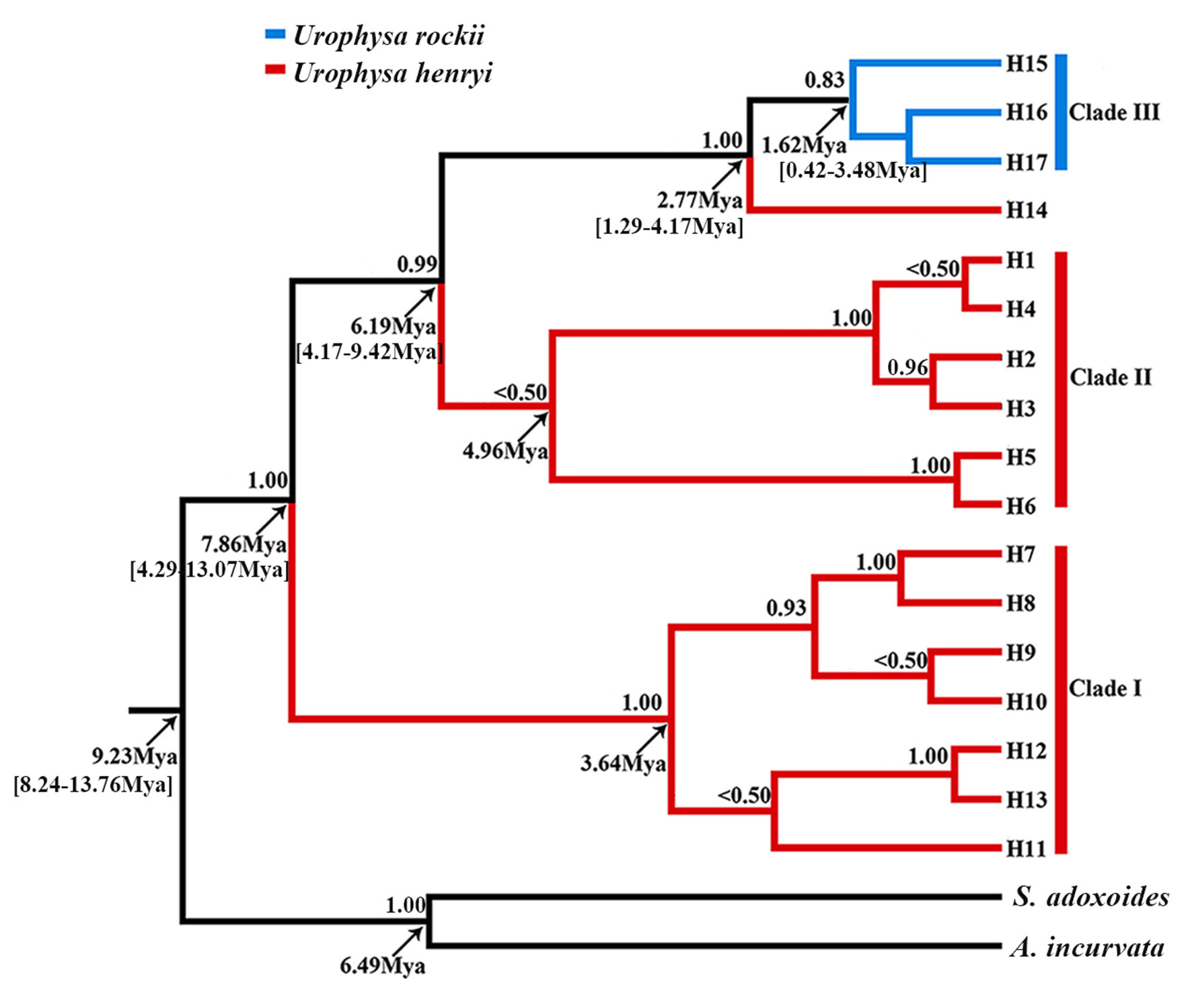

Supplement: S5 Fig — Numbers on the branches indicate the Bayesian posterior probabilities values. Ages of the main clades are shown below the branches. Different colors represent different haplotypes of species: blue, the haplotypes of U. rockii; red, the haplotypes of U. henryi. (TIF) [file pone.0186378.s005.tif]

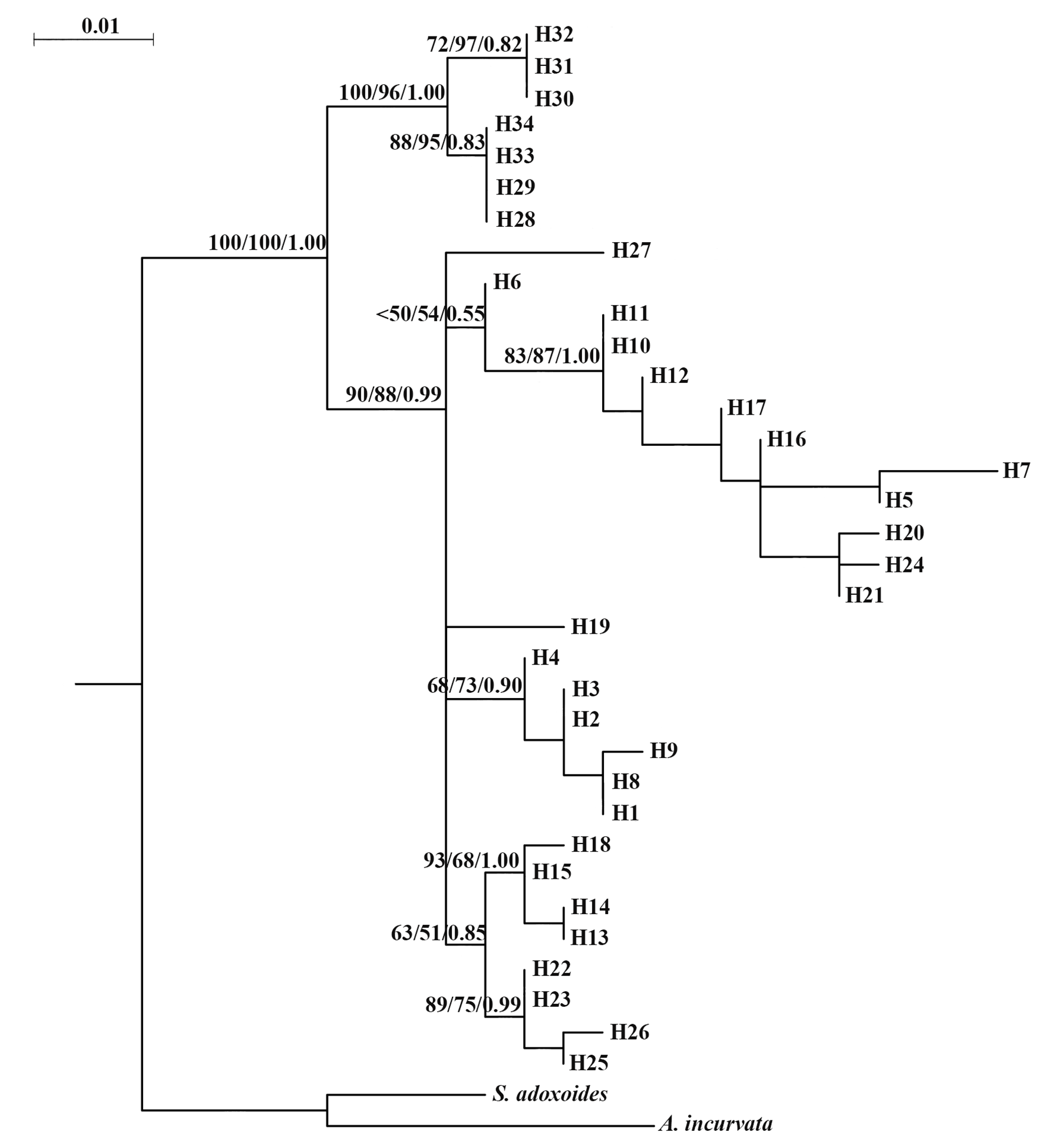

Supplement: S6 Fig — Numbers on the branches indicate the maximum Parsimony bootstrap, maximum likelihood support value and Bayesian posterior probabilities, respectively. (TIF) [file pone.0186378.s006.tif]

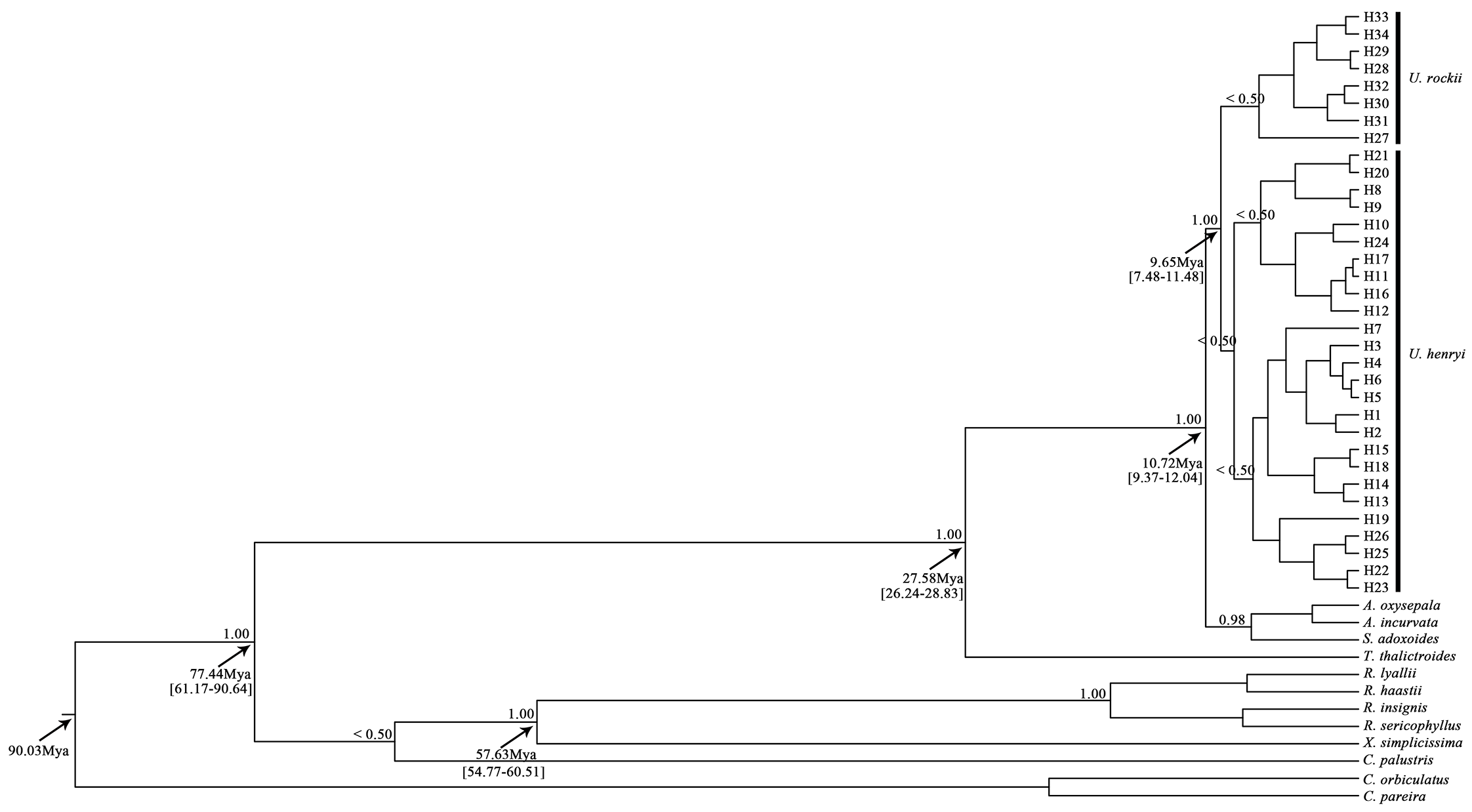

Supplement: S7 Fig — Numbers on the branches indicate the Bayesian posterior probabilities values. Ages of the main clades are shown below the branches. Haplotypes H1–H27 and H28–H34 are possessed by U. henryi and U. rockii, respectively. (TIF) [file pone.0186378.s007.tif]

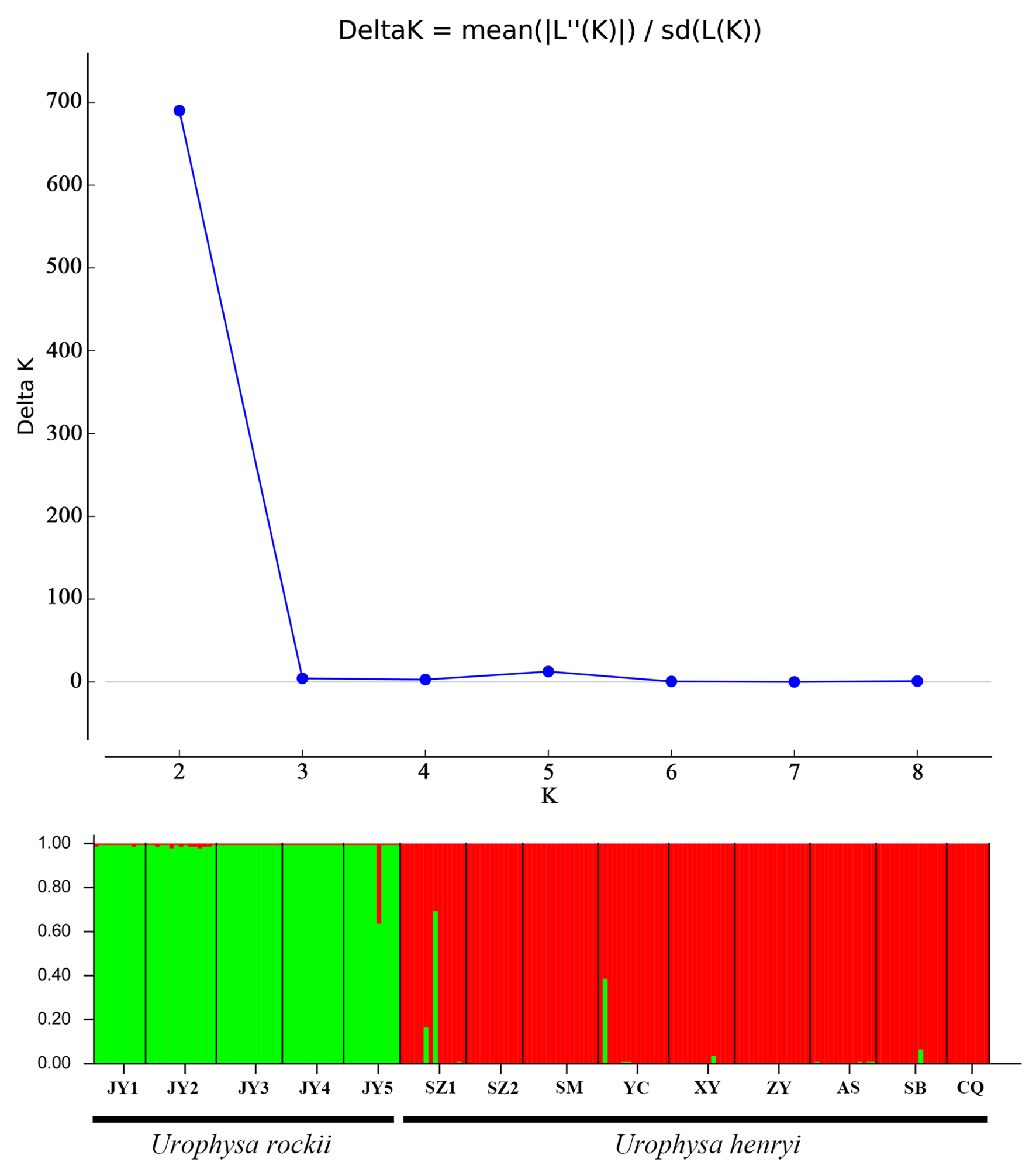

Supplement: S8 Fig — (a) Bayesian inference of the cluster number (K). (b) Results for clusters (K = 2) as detected by STRUCTURE. The bars on the figure represent these individuals that were retrieved from the same species. Bar plots showing Bayesian assignment probabilities. Each vertical bar corresponds to one individual. Populations are separated by black bars and identified at the bottom. (TIF) [file pone.0186378.s008.tif]
