# Supplementary material for: Phylogeography and genetic effects of habitat fragmentation on endemic Urophysa (Ranunculaceae) in Yungui Plateau and adjacent regions
Source: PLoS One. 2017 Oct 20;12(10):e0186378. doi: 10.1371/journal.pone.0186378 (PMC5650156; doi:10.1371/journal.pone.0186378)
Supplement: S1 Table — Numbers in the brackets is the sequence number of each haplotype included. *: represent the specific haplotype of each population. (DOC) [file pone.0186378.s009.doc]

**Table S1** Information of our haplotype sequences deposited in the GenBank.

| **Gene** | **GenBank numbers** | **Sequences** | **Number** |
| --- | --- | --- | --- |
| **EST** | KR820593-KR820627 | H1*(2), H2*(2), H3*(3), H4*(2), H5*(2), H6*(2), H7*(1)H8*(8), H9*(6), H10*(2), H11*(1), H12*(2), H13*(1), H14*(2), H15*(3), H16*(1), H17*(2), H18*(1), H19*(15), H20*(9), H21*(3), H22*(3), H23*(13), H24*(14), H25*(13), H26*(3), H27*(9), H28*(2), H29*(9), H30*(11), H31*(2), H32*(2), H33(14), H33(13), H34*(12) | 38 |
| **ITS** | KR820593-KR820628 |  | 38 |
| **pabA-trnH** | KR820665-KR820683 | H1(14), H1(8), H2*(2), H3(4), H1(8), H3(4), H4*(3), H5*(12), H6*(3), H7*(10), H8*(2), H9*(11), H10*(5), H11*(14), H12*(13), H13*(3), H14*(9), H15(11), H15(12), H16*(3), H15(14), H15(13), H17*(12) | 19 |
| **trnL-trnF** | KR820684-KR820702 |  | 19 |

Numbers in the brackets is the sequence number of each haplotype included. * : represent the specific haplotype of each population.
