# Supplementary material for: Phylogeography and genetic effects of habitat fragmentation on endemic Urophysa (Ranunculaceae) in Yungui Plateau and adjacent regions
Source: PLoS One. 2017 Oct 20;12(10):e0186378. doi: 10.1371/journal.pone.0186378 (PMC5650156; doi:10.1371/journal.pone.0186378)
Supplement: S2 Table — For each primer pair, forward (F) and reverse (R) primer sequence, repeat motif (Repeat), size of cloned allele (bp), optimal PCR annealing temperature (Ta). (DOC) [file pone.0186378.s010.doc]

**Table S2 Microsatellite markers used in this study.**

| **Marker** | **Primer sequence (5’-3’)** | **Size (bp)** | **Repeat motif** | **Ta/°C** |
| --- | --- | --- | --- | --- |
| **A41** | **F: CCAGTTCAGAAGACTTATCCCA** | **145** | **(TG)_3_(TA)_5_(TG)_4_** | **56** |
|  | **R: TTCAAATCACCCAGACACGA** |  |  |  |
| **B8** | **F: TAATGCTGTGCGGTTGTT** | **188** | **(AC)_11_** | **56** |
|  | **R: CCGAAATGGATGTTCCTTC** |  |  |  |
| **B21** | **F: TGTGGGTATGGCAAATGTGT** | **189** | **(GT)_7_** | **56** |
|  | **R: TATCCTCCTGCAAAACTGTG** |  |  |  |
| **EST1** | **F: AGTCGCAAATCTAACAAACG** | **346** | **(AC)_10_** | **52** |
|  | **R: CAGGGAAGGAAGAAAACATG** |  |  |  |
| **EST2** | **F: CCTTGCTTCCAAATGTGCTC** | **180** | **(CA)_9_** | **54** |
|  | **R: GGAGGAAATGCTGTTCTAATCG** |  |  |  |
| **EST3** | **F: TTTCAGCTAATTTTGGCGGC** | **227** | **(AC)_5_(TC)_11_** | **52** |
|  | **R: CGTAGTGTTGGATAGCAGTA** |  |  |  |
| **EST5** | **F: GCTGGATTTCACAGAAAGATAC** | **314** | **(CTT)_3_…****(TG)_6_** | **52** |
|  | **R: CCTTACAGTGATGACAACGA** |  |  |  |
| **EST8** | **F: CGCACTATTCCAGCTCATTC** | **211** | **(CT)_12_** | **54** |
|  | **R: GGGAACTTAAGCTCTTGAGG** |  |  |  |
| **EST9** | **F: GCTGCATTCCTCACATACTA** | **147** | **(GT)_5_** | **52** |
|  | **R: CATACATACGAAATCGGGTC** |  |  |  |

For each primer pair, forward (F) and reverse (R) primer sequence, repeat motif (Repeat), size of cloned allele (bp), optimal PCR annealing temperature (Ta).
