# Supplementary material for: Phylogeography and genetic effects of habitat fragmentation on endemic Urophysa (Ranunculaceae) in Yungui Plateau and adjacent regions
Source: PLoS One. 2017 Oct 20;12(10):e0186378. doi: 10.1371/journal.pone.0186378 (PMC5650156; doi:10.1371/journal.pone.0186378)
Supplement: S3 Table — (DOC) [file pone.0186378.s011.doc]

**Table S3** Homology test of each SSR locus between *Urophysa* and *Aquilegia*.

**A41**

>*Urophysa-*1.seq

TTTCAAATCACCCAGACACGAATTGGTTTCCTTCCGATTTCATTGAGCAGGGCCACATATACCACACACACACACACATATATATACACAT-CAATCTGACATGCAGAACTTATGAAGATAAATTGGGATAAGTCTTCTGAACTGGA

>*Urophysa-*2.seq

TTTCAAATCACCCAGACACGAATTGGTTTCCTTCCGATTTCATTGAGCAGGGCCACATATACCACACACACACACACATATATATACACA--CAATCTGACATGCAGAACTTATGAAGATAAATTGGGATAAGTCTTCTGAACTGGA

>*Aquilegia*

CTTCAAATCACCCAGACACGAATTGGTTTCCTTCCGATTTCATTGAGCAGGGCCACATATACCACACACGCACACACATATATATATACACACAATCTGACATGCAGAACTTATGAAGATAAATTGGGATAAGTCTTCTGAACTGGT

**B8**

>*Urophysa-*1.seq

CCGAAATGGATGTTCCTTCAAAATGCAGATAATATGGGGTGTGTGTGTGTGTGTGTGTGTGTTTCTATCAAAATACACACATAACTGATTAACCAGATAAAAAAAGTACCTTCTTGAGCCGACAT-CAGAAATACTGAGCCAAGTATCAACGCACAAAATGTAGTGAAGAACAACCGCACAGCATTAA

>*Urophysa-*2.seq

CCGAAATGGATGTTCCTTCAAA-TGCAGATAATATGG--TGTGTGTGTGTGTGTGTGGGTGTTTCTATCAAAATACACAAATAACTGATTAACCAGATAAAAAAAGTACCTTCTTGAGCCGACATTCAGAAATACTGAGCCAAGTATCAACGCACAAAATGTAGTGAAGAACAACCGCACAGCATTAA

>*Aquilegia*

CCGAAATGGATGTTCCTTCAAAATGCAGATAATATGG------TGTGTGTGTGTGTGTGTGTGTCTATCAAAATACACACATAACTGATTAGCCAGATAAAAAAAGTACCTTCTTGAGCCGACATTCAGAAATACTGAGCCAAGTATCAATGCACAAAATGTAATGAAGAACAACCGCACAGCATTAT

**B21**

>*Urophysa-*1.seq

ATTATATATTATTGGACCAACCCCGTTTTTCCGGAAGAAAAAAATATAATTCATGGCATCTCAAAGTCTCAACATTATATCTCAATCACTACAAATCAATCATTGCATCCAAACACACTTCACAACCTATACCCAATCACTAGTACACCTCCCACAGGATGCACTCCACACATTTGCCATACCCACAAA

>*Urophysa-*2.seq

ATTATATATTATTGGACCAACCCCGTTTTTCCGGAAGAAAAAAATATAATTCATGGCATCTCAAAGTCTCAACATTATATCTCAATCACTACAAATCAATCATTGCATCCAAACACACTTCACAACCTATACCCAATCACTAGTACACCTCCCACAGGATGCACTCCACACATTTGCCATACCCACAAA

>*Aquilegia*

TTGACCTATCCTCCTGCAAA----ACTGTGTTAGTACAATAATATATAATTCATGGCATCTCAA-GTCTCGACACTATATCTCAATCACTACAAATCAATCATTACACACACACACACTTCACAACCTATACCCAATCACTAGTACACCTCCCACAGGATGCACTCCACACATTTGCCATACCCACACT

**EST1**

>*Urophysa*-1.seq

AGGAATCAGGGAAAGA-GAAAACATGGACAGAGAAGATATAAAGATGTTAGTGAAGAAATGGTGGGACATTTACAATGACGCTTCACTTGATTACAAGAGGAGTGTTGCTCCTTCAATTCCAGAGACTGGTGAAAATCTTCAACCATTTCTGGCTGCTTTATCTGAAGCTGGAGTGGTTCACTATGTTACTGCTCCATCGGCCGCATGATGCATGGACCATCTGCTGTGCTGATTACTTAGTGTGTGTGTGTGTGTG----AAGTTATAATGCAGTATTAAAGTAATAGAATAGAAATTGTATACAGGCAGATGGTCGCAATTTTCTGTTTTCGTTTGTTACATTTTTCGACTAGT

>*Urophysa-*2.seq

-----TCAGGGAAGGAAGAAAACATGGACAGAGAAGACATAAAGATGTTAGTGAAGAAATGGTGGGACATTTACGATGACTCTTCACTTGATTACAGAAGGAGTGTTGCTCCTTCAATTCCAGAGACTGGTGAAAATCTTCAACCATTTCTGGCTGCTTTATCTGAAGCTGGAGTGGTTCACTATGTTACTGCTCCATCGGCCGCATGATGAATGGACCATCTGCTGTGCTGATTACTTAGTGTGTGTGTGTGTGTG----AAGTTATAATGCAGTATTAATGTAATAGAATAGAAATTGTATACAGGCAGATGGTCGCAATTTTCTGTTTTCGTTTGTTAGATTTTTCGACTA--

>*Aquilegia*

GGTATACAGGGAAGGAAGAAAACATGGACAGAGAAGATATAAAGATGTTAGTGAAGAAATGGTGGGACATTTACGATGACGCTTCACTTGATTACAAGAGGAGTGTTGCTCCTTCAATTCCAGAGACTGGTGAAAACCTTCAACCATTTCTGGCTGCTTTATCTGAAGCTGGAGTGGTTCACTATGTTACTGCTCCATCGGCCGCATGATGCGTGGACCATCTGCTGTGCTGATTACTGAGTGTGTGTGTGTGTGTGTGTGAAGTTATAATGCAGTATTAATGTAATAGAATAGAAATTGTATACAGGCAGATGGTCTCAAATTTCTGTTTTCGTTTGTTAGATTT-GCGACTTGG

**EST2**

>*Urophysa*-1.seq

TGGAGGAAATGCT-TTCTAATCGATCCATCTTGGAGTAGCTTTCCATTCAACAGTGAAGATTTTGGCATGGTGCTTATAGAATAAAGTCCGATTTTTGTGTGTGTGTGTG--------CAGATCAAGTTCAAAGAAGCTGAATAGAGGGCCATCTTTTAAAGCCTAGAGCACATTTGGAAGCAA---

>*Urophysa-*2.seq

TGGAGGAAATGCT-TTCTAATCGATGCATCTTGGAGTAGCTTTCCATTCAACAGTGAAGATTTTGGCATGGTGCTTATAGAATAAAGTCCGATTTGTGTGTGTGTGTGTGTG------CAGATCAAGTTCAAAGAAGCTGAATAGAGGGCTATCTTTTAAAGCCTAGAGCACATTTGGAAGCAAGGA

>*Aquilegia*

GGGAGGAAATGCTGTTCTAATCGATGCATCTTGGAGTAGCTTTCCATTCAACAGTGAAGATTTTGGCATGGTGCTTATAGAATAAAGTCCGATTTGTGTGTGTGTGTGTGTGGGTTTGCAGATCAAGTTCAAAGAAGCTGAATAGAGGGCCATCTTTTAAAGCCTAGAGCACATTTGGAAGCAAGGA

**EST3**

>*Urophysa*-1.seq

TCGTAGTGTTGGATAGCAGTAAGAGGCTGCTGCT------TCTGTTGCAATAGCATCATCTTCCTTTTCTTCTTTCTCTCCATTGGTTTTACAAGTTAACATTTCATCTTCTTGCTCTTCTTCTCCCATTTGTGTTTACTGTGATTGAAAGAGAGAG----------------TGTGTGTGTCACTATTGT--------GTTCCTGTTACTGACTCACGAGTGACATTTTGCCGCCAAA-TTAGCTGAA

>*Urophysa-*2.seq

TCGTAGTGTTGGATAGCAGTAAGAGGCTGCTGCT------TCTGTTGCAATAGTATCATCTTCCTTTTCTTCTTTCTCTCCATTGGTTTTACAAGTTAACATTTCATCTTCTTGCTCTTCTTCTCCCATTTGTGTTTACTGTTATTGAAAGAGAGAAA----------------GTGTTTGTCACTATTGTCGTATTGTGTTCCTGTTAGTGACTCACGAATGACATTTTGCCGCCAAA-TTAGCTGAA

>*Aquilegia*

ACGTAGTGTTGGATAGCAGTAAGGGGCTGCTGCTGCTGCTTCTGTTGCAATAGCATCATCTTCCTTTTCTTCTTTCTCTCTATTGGTTTTACAAGTTACCATTTCATCTTCTTGCTCTTCTTCTCCCATTTGTGTTTACTGTTATTGAAAGAGAGAGAGAGAGAGAGAGAGAGTGTGTGTGTCACTATTGTAGTATTGTGTTCTTGTTACTGACTCACGAGTGACATTTTGCCGCCAAAATTAGCTGAA

**EST5**

>*Urophysa*-1.seq

GACAACGAAAACAAGCCTTATGATAAACTTTGTTATCAGCAGTAAGCTGATCAACTAAGTAAACAGTTTTCTCACAAGCTTTGCATTTCTGAGTTGTTCCAGCAAAAGTTGCCATTTTCAGATCTCTATTTTTACTTCTCTTTTGAACAAGAGTATCACACACACACACACAGATGACAGACCCAAAGTTGTATTATAAAAGCACAGAAGAAAGTGAAGATGAAGAAGA---TTGAAAGATTCTTGTTTTAGTGAAAAAGCAAAGAAGAT-GACAAATGTTGATGAAGAGCTGACTCTGTTTGTATCTTTCTGGTAAA

>*Urophysa-*2.seq

GACAACGAAAACAAGCCTTATGATAAACTTTGTTATCAGCAGTAAGCTGATCAACTAAGTAAACAGTTTTCTCACAAGCTTTGCATTTCTGAGTTGTTCCAGCAAAAGTTGCCATTTTCAGATCTCTATTTTTACTTCTCTTTTGAACAAGAGTATCACACACACACACACAGATGACAGACCCAAAGTTGTATTATAAAAGCACAGAAGAAAGTGAAGATGAAGAAGA---TTGAAAGATTCTTGTTTTAGTGAAAAAGCAAAGAAGAT-GACAAATGTTGATGAAGAGCTGACTCTGTTTGTATCTTTCTGGTAAA

>*Aquilegia*

GACAACGAAAACAAGCCTTATGATAAACTTTGTTATCAGCAGTAAGCTGATCAACTAAGTAAACAGTTTTCTCACAAGCTTTACATTTCTGAGTTGTTCCAGCAAAAGTAGCCATTTTCAGATCTCTATCTTTACTTCTCTTTTGAACAAGAGTATCACACACACAC----AGATGACAGACCC--AGTTGTTATATAAAAGCAGAGAAGAAAGTGAAGATGAAGAAGAAGATTGAAAGATTCTTGTTTTAGTGAAAAAGCAAAGAAGATTGACAAATGTTGATGAAGAGCTGACTCAGTTTGTATCTTTCTGTGAAA

**EST8**

>*Urophysa*-1.seq

TGTGAACTTGTGATCTTAAGCTCTTGCGGTGCGACACTGTGAACCTCTACACCTGAAGAACTCATTTCTTGATCCAATTAAGCGATCAATTGGATTTCAAACGAAGATCTGGAGTGTTAAAGATTCTGGTTTTTGGGAAGGTTAAGAGTGAATAATACAAT--CTCAGAAGAGAGAGAGAGAGAGAGAGAGAGAGAGAGAAAGAGATGTGCGA

>*Urophysa-*2.seq

--------TGGGAACTTAAGCTCTTGAGGTTCAACAGTGAGAAGCTCTACACCTGAG--ACTCATTTCTTGATCCAATTAAGCGATCAATTGGATTTCAAACGAAGATCTGGAGTGTTAAAGATTCTGGTTTTTGGGAAGGTTAAGAGTGAATAATACAAT--CTCAGAAGAGAGAGAGAGAGAGAGAGAAAGA----GCTGGAATAGTGCGA

>*Aquilegia*

AATTCAAAAGGGAACTTAAGCTCTTGAGGTTCAACAGTGAGAAGCTCTCCACCTGAAGAACTCATTTCTTGATCCAATTAAGCGATCAATTGGATTTCAAACGAAGATCTGGAGTGTTAAAGATTCTGGTTTTTGGAAAGGTTGAGAGTGAATAATACAATTTCTCAGAAGAGAGAGAGAGAGAGAGAGAGAGAATGAGCTGGAATAGTGCGC

**EST9**

>*Urophysa*-1.seq

TCATACATACGAAATCGGGTCAGTTTGGAAAAACATTGTACTGTCACCTACAAGGACACACATACAAGACGAGCTAAAACTTTCCAT-CAAAAATCAGAGAGTCTAGCCGCAACAAATCTATAGCAATTAGTATGTGAGGAATGCAGC

>*Urophysa-*2.seq

TCATACATACGAAATCGGGTCAGTTTGGAAAAACATTGTACTGTCACCTACAAGGACACACATACAAGACGAGCTAAAACTTTCCAT-CAAAAATCAGAGAGTCTAGCCGCAACAAATCTATAGCAATTAGTATGTGAGGAATGCAGC

>*Aquilegia*

CCATACATACGAAATCGGGTCAGTTTGGAAAAACATTGTACTGTCACCTACAAGGACACACACACAAGACGAGCTAAAACTTTCCATTCAAAAATCAGAGAGTCTAGCCGCGACAAATCTATAGCAATTAGTATGTGAGGAATGCAGC
