# Supplementary material for: Phylogeography and genetic effects of habitat fragmentation on endemic Urophysa (Ranunculaceae) in Yungui Plateau and adjacent regions
Source: PLoS One. 2017 Oct 20;12(10):e0186378. doi: 10.1371/journal.pone.0186378 (PMC5650156; doi:10.1371/journal.pone.0186378)
Supplement: S4 Table — (DOC) [file pone.0186378.s012.doc]

**Table S4** psbA-trnH and trnl-trnF sequences of outgroups from Genbank for time calibration.

| **Species** | **GeneBank numbers** | |
| --- | --- | --- |
|  | **psbA-trnH** | **trnL-trnF** |
| ***Aquilegia oxysepala*** | KY235737 | EF437097 |
| ***Thalictrum thalictroides*** | KP643250 | JX573511 |
| ***Ranunculus lyallii*** | FJ744179 | FJ744235 |
| ***Ranunculus haastii*** | FJ744173 | AB617680 |
| ***Ranunculus insignis*** | FJ744176 | AB617680 |
| ***Ranunculus sericophyllus*** | FJ744181 | AB617680 |
| ***Xanthorhiza simplicísima*** | AB163750 | AM397155 |
| ***Caltha palustris*** | KP643478 | AY365376 |
| ***Cocculus orbiculatus*** | GQ434992 | EF143891 |
| ***Cissampelos pareira*** | KJ426656 | KX384122 |
