# Supplementary material for: Phylogeography and genetic effects of habitat fragmentation on endemic Urophysa (Ranunculaceae) in Yungui Plateau and adjacent regions
Source: PLoS One. 2017 Oct 20;12(10):e0186378. doi: 10.1371/journal.pone.0186378 (PMC5650156; doi:10.1371/journal.pone.0186378)
Supplement: S6 Table — NM: gene flow. (DOC) [file pone.0186378.s014.doc]

**Table S6** Parameters of Neutrality test and gene flow among populations.

| **Populations** | **Tajima's D** | ***P*** | **Fu and Li's D*** | ***P*** | **Fu and Li's F*** | ***P*** | ***N*_M_** |
| --- | --- | --- | --- | --- | --- | --- | --- |
| **cpDNA** |  |  |  |  |  |  |  |
| *U. rockii* | 0.248 | >0.10 | –0.316 | >0.10 | –0.159 | >0.10 | 0.03 |
| *U. henryi* | 1.147 | >0.10 | 0.917 | >0.10 | 1.213 | >0.10 | 0.01 |
| Clade I | 0.182 | >0.10 | –1.283 | >0.10 | –1.082 | >0.10 | 0.03 |
| Clade II | 1.892 | >0.05 | 1.314 | >0.05 | 1.787 | <0.05 | 0.01 |
| **nrDNA** |  |  |  |  |  |  |  |
| *U. rockii* | 1.453 | >0.10 | 0.784 | >0.10 | 1.183 | >0.10 | 0.01 |
| *U. henryi* | –0.206 | >0.10 | 0.343 | >0.10 | 0.146 | >0.10 | 0.05 |

***N*_M_**: gene flow.
