# Supplementary material for: Phylogeography and genetic effects of habitat fragmentation on endemic Urophysa (Ranunculaceae) in Yungui Plateau and adjacent regions
Source: PLoS One. 2017 Oct 20;12(10):e0186378. doi: 10.1371/journal.pone.0186378 (PMC5650156; doi:10.1371/journal.pone.0186378)
Supplement: S7 Table — Numbers on the branches indicate the maximum likelihood support value and Bayesian posterior probabilities, respectively. Different colors represent different populations of species: red, the populations of U. henryi; blue, the populations of U. rockii. (DOC) [file pone.0186378.s015.doc]

**The substitution model of each locus is:**

**nrDNA:** ITS: the substitution model is GTR+T

ETS: the substitution model is TVM+G

**cpDNA:** psbA-trnH: the substitution model is HKY+G

trnL-trnF: the substitution model is K81uf+I


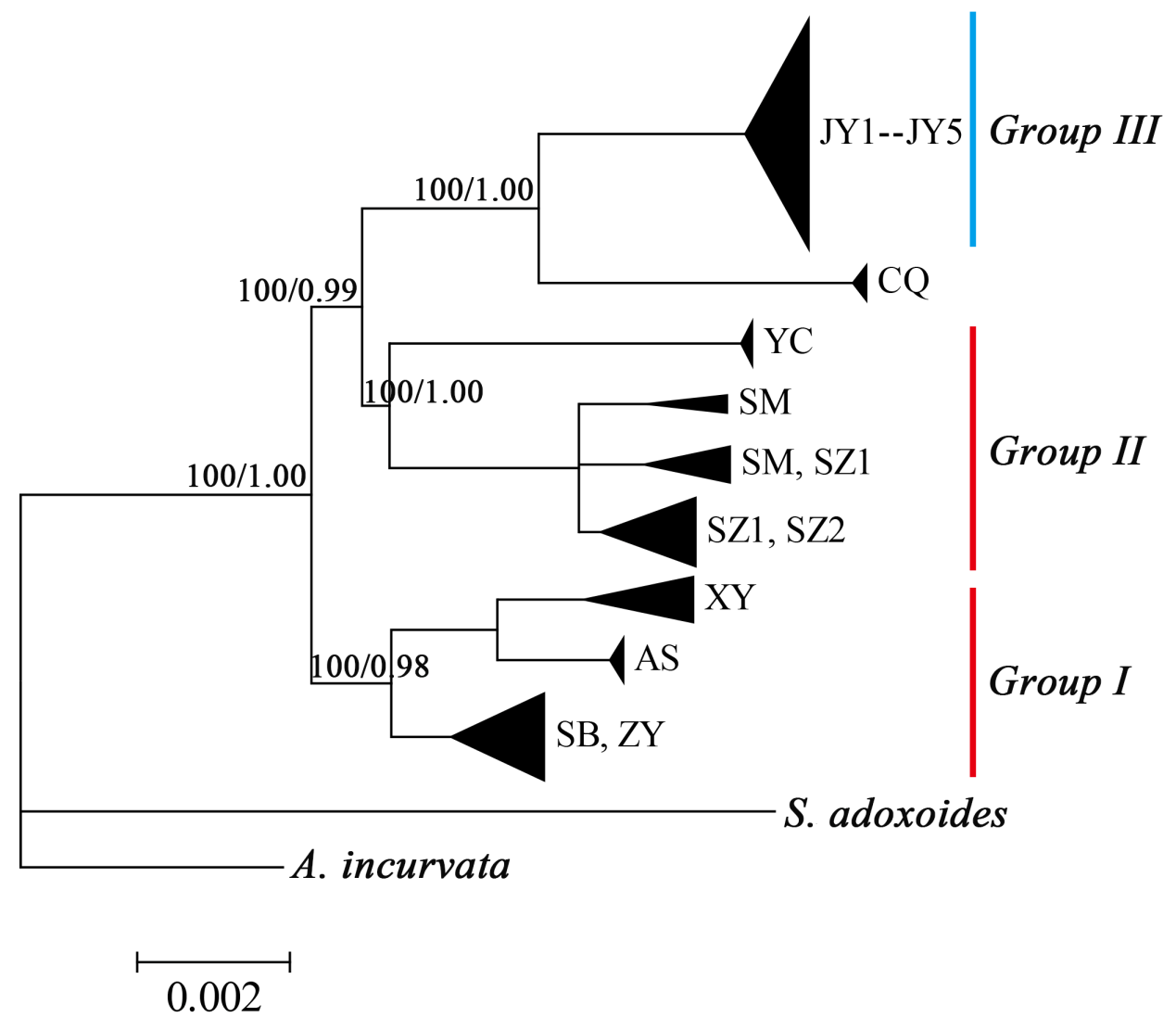


**Table S7 The phylogenetic analysis both on RAxML and MrBayes based on substitution model of each locus.** Numbers on the branches indicate the maximum likelihood support value and Bayesian posterior probabilities, respectively. Different colors represent different populations of species: red, the populations of *U. henryi*; blue, the populations of *U. rockii*.
