# Supplementary material for: Phylogeography and genetic effects of habitat fragmentation on endemic Urophysa (Ranunculaceae) in Yungui Plateau and adjacent regions
Source: PLoS One. 2017 Oct 20;12(10):e0186378. doi: 10.1371/journal.pone.0186378 (PMC5650156; doi:10.1371/journal.pone.0186378)
Supplement: S9 Table — Note: Values in bold were not significantly different from zero after sequential Bonferroni correction.*: significant at p < 0.001. (DOC) [file pone.0186378.s017.doc]

**Table S9** Pairwise F_ST_ values among the 14 populations of *U. henryi* and *U. rockii* based on SSRn data*.*

|  | JY1 | JY2 | JY3 | JY4 | JY5 | SZ1 | SZ2 | SM | YC | XY | ZY | AS | SB | CQ |
| --- | --- | --- | --- | --- | --- | --- | --- | --- | --- | --- | --- | --- | --- | --- |
| JY1 | 0.0000 |  |  |  |  |  |  |  |  |  |  |  |  |  |
| JY2 | 0.3136* | 0.0000 |  |  |  |  |  |  |  |  |  |  |  |  |
| JY3 | 0.3625* | 0.4161* | 0.0000 |  |  |  |  |  |  |  |  |  |  |  |
| JY4 | 0.2365* | 0.3984* | **0.0542** | 0.0000 |  |  |  |  |  |  |  |  |  |  |
| JY5 | 0.2165* | 0.4068* | 0.2235* | 0.1237* | 0.0000 |  |  |  |  |  |  |  |  |  |
| SZ1 | 0.4936* | 0.4403* | 0.5478* | 0.5507* | 0.5054* | 0.0000 |  |  |  |  |  |  |  |  |
| SZ2 | 0.6063* | 0.5589* | 0.6343* | 0.6390* | 0.6049* | 0.3417* | 0.0000 |  |  |  |  |  |  |  |
| SM | 0.5852* | 0.5285* | 0.6182* | 0.6232* | 0.5847* | 0.3177* | 0.4707* | 0.0000 |  |  |  |  |  |  |
| YC | 0.5825* | 0.5101* | 0.5757* | 0.5834* | 0.5506* | 0.3267* | 0.4331* | 0.3497* | 0.0000 |  |  |  |  |  |
| XY | 0.6684* | 0.5565* | 0.6621* | 0.6761* | 0.6449* | 0.4732* | 0.5230* | 0.5399* | 0.4462* | 0.0000 |  |  |  |  |
| ZY | 0.5371* | 0.4674* | 0.5698* | 0.5733* | 0.5431* | 0.4187* | 0.4886* | 0.4732* | 0.4403* | 0.5206* | 0.0000 |  |  |  |
| AS | 0.5923* | 0.4927* | 0.5796* | 0.5902* | 0.5530* | 0.3954* | 0.4396* | 0.4419* | 0.4191* | 0.3365* | 0.4820* | 0.0000 |  |  |
| SB | 0.5638* | 0.5432* | 0.5893* | 0.5881* | 0.5621* | 0.3891* | 0.4029* | 0.4169* | 0.4617* | 0.4856* | 0.4872* | 0.3483* | 0.0000 |  |
| CQ | 0.6010* | 0.4890* | 0.5980* | 0.6111* | 0.5768* | 0.4003* | 0.4444* | 0.4518* | 0.3816* | 0.3015* | 0.4750* | 0.1695* | 0.3962* | 0.000 |

Note: Values in bold were not significantly different from zero after sequential Bonferroni correction.*: significant at *p* < 0.001
